# Association of influenza viral genetic information with severity markers in patients hospitalised with influenza: multicentre retrospective cohort study

**DOI:** 10.1136/bmjopen-2025-111643

**Published:** 2026-01-08

**Authors:** Aung Pone Myint, George Shirreff, Vicky Baillie, Antonin Bal, Celina F Boutros, Elena Burtseva, Daouda Coulibaly, Daria Danilenko, Ghassan Dbaibo, Gregory Destras, Ndongo Dia, Anca Cristina Drăgănescu, Heloisa I G Giamberardino, Andrey B Komissarov, Parvaiz A Koul, Victor Alberto Laguna-Torres, Jason J LeBlanc, Ainara Mira-Iglesias, Alla Mironenko, Alejandro Orrico-Sánchez, Nancy A Otieno, Oana Săndulescu, Viviana Simon, Anna Sominina, Emilia Sordillo, Mine Durusu Tanriover, Nataliia Teteriuk, Serhat Unal, Harm Van Bakel, Melissa K Andrew, Joseph Bresee, Bruno Lina, F Xavier López-Labrador, Justin R Ortiz, Sonia M Raboni, Wenqing Zhang, Sandra S Chaves, Giacomo Cacciapaglia, Laurence Josset, Cécile Chauvel, Marta C Nunes

**Affiliations:** 1 Université Claude Bernard Lyon 1, Villeurbanne, France; 2 Centre of Excellence in Respiratory Pathogens (CERP), Hospices Civils de Lyon (HCL) and Centre International de Recherche en Infectiologie (CIRI), Équipe Santé Publique, Épidémiologie et Écologie Évolutive des Maladies Infectieuses (PHE3ID), Inserm U1111, CNRS UMR5308, ENS de Lyon, Université Claude Bernard Lyon 1 (UCBL Lyon 1), Lyon, France; 3 South African Medical Research Council, Vaccines and Infectious Diseases Analytics (VIDA) Research Unit, Faculty of Health Sciences, University of the Witwatersrand, Johannesburg, South Africa; 4 HCL and CIRI, Inserm U1111, CNRS UMR5308, ENS de Lyon, UCBL Lyon 1, Lyon, France; 5 American University of Beirut Centre for Infectious Diseases Research, Beirut, Beirut Governorate, Lebanon; 6 Gamaleya National Research Centre for Epidemiology and Microbiology, Ministry of Health of Russian Federation, Moscow, Russian Federation; 7 Institut National d’Hygiène Publique, Abidjan, Côte d’Ivoire; 8 Smorodintsev Research Institute of Influenza, Saint Petersburg, Russian Federation; 9 Centre for Infectious Diseases Research, American University of Beirut, Beirut, Lebanon; 10 Institut Pasteur of Dakar, Dakar, Senegal; 11 National Institute of Infectious Diseases Prof Dr Matei Bals, Bucharest, Romania; 12 Hospital Pequeno Principe, Curitiba, Paraná, Brazil; 13 Sher-i-Kashmir Institute, Srinagar, Jammu and Kashmir, India; 14 Clínica Internacional, Instituto de Medicina Tropical, Universidad Nacional Mayor de San Marcos, Lima District, Lima Region, Peru; 15 Dalhousie University, Halifax, England, Canada; 16 FISABIO, Valencia, Valencian Community, Spain; 17 CIBERESP, Instituto de Salud Carlos III, Madrid, Community of Madrid, Spain; 18 SI Kyiv City Centre for Diseases Control and Prevention of the Ministry of Health of Ukraine, Kyiv, Ukraine; 19 Kenya Medical Research Institute, Kisumu, Kenya; 20 Carol Davila University of Medicine and Pharmacy, National Institute for Infectious Diseases “Prof. Dr. Matei Balș”, Bucuresti, Bucuresti, Romania; 21 Icahn School of Medicine at Mount Sinai, New York, New York, USA; 22 Vaccine Institute, Hacettepe University, Ankara, Ankara, Turkey; 23 Department of Infectious Diseases and Clinical Microbiology, Hacettepe University School of Medicine, Ankara, Turkey; 24 Partnership for International Vaccine Initiatives, The Task Force for Global Health, Decatur, Georgia, USA; 25 Department of Microbiology, Medical School, University of Valencia, Valencia, Valencian Community, Spain; 26 Centre for Vaccine Development and Global Health, University of Maryland Baltimore School of Medicine, Baltimore, Maryland, USA; 27 Molecular Biology/Microbiology Research Laboratory, Universidade Federal do Paraná, Curitiba, Paraná, Brazil; 28 Global Influenza Program, World Health Organization, Geneve, Switzerland; 29 Foundation for Influenza Epidemiology, Fondation de France, Paris, Île-de-France, France; 30 Laboratoire de Physique Théorique et Hautes Energies (LPTHE), UMR 7589, Sorbonne Université and CNRS, Paris, France

**Keywords:** Respiratory infections, EPIDEMIOLOGY, Epidemiology, Epidemics, INFECTIOUS DISEASES

## Abstract

**Abstract:**

**Objective:**

The objective of this study was to determine the association between viral subtype/clade and disease severity.

**Design:**

Multicentre retrospective cohort study.

**Setting:**

This study used data from the Global Influenza Hospital Surveillance Network (GIHSN). The dataset comprised hospitalised influenza patients with viral sequencing data across 14 countries, collected from August 2022 through October 2023.

**Participants:**

A total of 761 hospitalised patients were enrolled during the study period, and 745 patients were included in the analysis. We excluded patients with missing data on explanatory or outcome variables, those infected with viral clades represented by fewer than 11 sequences, and those enrolled at study sites contributing fewer than 5 patients.

**Outcome measures:**

Disease severity was defined by admission to intensive care unit (ICU), receipt of non-invasive oxygen supplementation, 3-variable definition (ICU, mechanical ventilation or death) or 4-variable definition (3-variable plus oxygen supplementation).

Outcomes were analysed in association with subtype or clade using the mixed-effects logistic regression models, adjusting for age group, sex, underlying medical conditions, influenza vaccination status, antiviral use, country income level and epidemic period, while study site was included as a random effect.

**Results:**

745 patients were included: 263 A(H1N1)pdm09, 380 A(H3N2), 102 B/Victoria. A(H1N1)pdm09 infection was associated with increased odds of ICU admission (adjusted ORs (aORs) 2.5, 95% CI 1.1 to 5.8) compared with A(H3N2). 6B.1A.5a.2a.1 clade of A(H1N1)pdm09 was associated with increased severity compared with 6B.1A.5a.2a clade (aOR 3.0, 95% CI 1.0 to 9.5) and (aOR 5.4, 95% CI 1.6 to 18.3) for the 3-variable and 4-variable definitions respectively. Among A(H3N2), the (3C.2a1b.2a.)2b clade showed a trend toward increased severity using the 4-variable definition compared with the 2a.1b clade (aOR 2.9, 95% CI 0.8 to 10.0).

**Conclusions:**

This analysis highlights the differential impact of influenza subtypes and clades on disease severity in hospitalised patients. Future research should investigate the role of specific viral mutations of these clades in modulating immune evasion or disease severity. These findings reinforce the GIHSN’s critical role in global surveillance. Ongoing genomic surveillance is crucial for understanding the clinical impact of emerging influenza variants and informing public health responses.

**STRENGTHS AND LIMITATIONS OF THIS STUDY**

- The study’s robust and comprehensive design, drawing on a geographically diverse patient population across multiple countries with varied socioeconomic statuses, enhances the generalisability and reliability of the findings.
- The random effects models were employed to account for variability between study sites, yielding more accurate and reliable estimates of the overall effect.
- The use of multiple outcomes effectively captures the full spectrum of disease severity, complemented by sensitivity analyses to confirm the stability of the main findings.
- Generalisability is limited as the cohort is restricted to hospitalised patients with viral genome sequencing data and may not represent the broader population with influenza infection.
- Site-specific surveillance bias may have been introduced by variations in hospital types and differing clinical indications across sites for interventions such as oxygen supplementation or intensive care unit admission.

## Introduction

 Influenza remains a major global health threat, accounting for considerable morbidity and mortality, particularly among the most vulnerable populations. It had been estimated that 3.2 million cases of hospitalisations had been associated with the seasonal influenza epidemics annually.^1^ Influenza’s impact extends beyond the respiratory system, contributing to exacerbations of cardiovascular and chronic respiratory illnesses,^2^ highlighting its extensive health burden, which varies annually depending on influenza vaccine coverage and effectiveness. Seasonal influenza is primarily caused by influenza A and B viruses. Influenza A viruses are subtyped according to their surface proteins: haemagglutinin (HA) and neuraminidase (NA), with A(H1N1) and A(H3N2) currently circulating in humans. Influenza B viruses are categorised into two lineages, B/Victoria and B/Yamagata, with B/Yamagata lineage not being detected since 2020.^3^ These viruses undergo continuous evolution through antigenic drift that gradually leads to the accumulation of mutations resulting in immune system evasion and causing annual epidemics, or antigenic shift, drastic viral changes that can cause pandemics.^4^

Recent studies highlight the higher rates of hospitalisations associated with A(H3N2) subtype compared with A(H1N1)pdm09 or influenza-B with some seasonal variations.^5 6^ Nonetheless, A(H1N1) has been associated with severe disease among those born after 1957^7^ and is often associated with more severe outcomes in hospitalised patients.^8^,^14^ Notably, patients infected with specific clades such as the A(H3N2) reassortant re3C.2a.2 had higher Influenza Severity Score during the particularly harsh 2017–2018 season in the USA,^15^ underscoring the need to track the rapid genomic changes and emergent strains of influenza viruses.

This study leverages data from the Global Influenza Hospital Surveillance Network (GIHSN), which encompasses a consortium of hospitals in more than 25 countries across diverse geographical regions.^16^ During the 2022–2023 season (defined by the GIHSN as November through October), the GIHSN collected data from over 30 000 hospitalised patients, identifying 3285 confirmed influenza cases.^17^ Notably, of these cases, 888 whole genome sequences (WGSs) were generated and shared via the EpiFlu database of the Global Initiative on Sharing All Influenza Data (GISAID) platform.^17^ Given the constant evolution of influenza viruses and emergence of new variants, understanding the viral genetic information associated with severe disease is crucial to inform public health strategies. By combining detailed clinical data with genomic sequencing, we aimed to identify specific viral clades that were associated with severe clinical outcomes, providing insights for risk assessment and the development of targeted interventions.

## Methods

The GIHSN enrolled patients admitted to hospitals for at least one night due to respiratory illness or symptoms indicative of recent respiratory infection.^18^ Healthcare professionals identified potential participants within 72 hours of admission based on disease presentation specific to each study site. Patients meeting these criteria and providing consent were enrolled and tested for influenza using PCR technique. Genome sequencing, preferably WGS, was performed at each study site or by sending the samples to the National Influenza Centre in Lyon, France, or to the WHO Collaborating Centres, using a harmonised sequencing protocol. These data were submitted to GISAID under unique identifiers linking back to the GIHSN metadata. Some sites selectively sequenced specimens from critical patients, like those admitted to intensive care units (ICUs), and these sites were not considered for the current analysis, prioritising the data from sites testing a variety of disease-severity patients or a random number of patients.

Patients included in the current analysis were those enrolled in the GIHSN who tested positive for influenza between August 2022 and October 2023, with available viral genomic sequence data on GISAID. This time frame was chosen to include patients from countries with different seasonal patterns. Patients were excluded from the main analysis if they had missing data on any explanatory variable (n=14), had incomplete severity information (varied depending on the outcomes, as detailed in tables 1–3), or were transferred to another hospital without a documented severe outcome before transfer (n=2). For clade-level analyses, sequences lacking clade information were excluded from the analysis. Furthermore, clades reported in 10 or fewer patients were excluded to ensure statistical reliability and model stability. For each model, study sites with fewer than five participants were omitted.

The primary outcome, disease severity, was assessed using available variables in the GIHSN dataset. Individual severity markers included the need for non-invasive oxygen supplementation (as measured at admission) and ICU admission. Composite severity definitions were defined in two ways: 3-variable composite, with patients who had at least one of mechanical ventilation, ICU admission or in-hospital death; and 4-variable composite, patients with 3-variable definition or oxygen supplementation. For individual severity markers, patients with missing data for the relevant marker were excluded. For composite definitions, participants were classified as severe if they were recorded as ‘yes’ for at least one of the criteria, regardless of missing information for the other criteria. Patients who had ‘no’ and missing information for at least one of the other criteria, as well as those with missing information for all criteria, were excluded from the main analysis.

Various demographic and clinical characteristics available in the dataset were summarised and included as explanatory variables in the model as they have been shown to be associated with disease severity. These included: age group (<5, 5–17, 18–64 and ≥65 years),^9 14^ presence of underlying medical conditions (cardiovascular diseases, chronic lung diseases, asthma, diabetes, immunodeficiency, HIV infection, organ transplant, renal impairments, rheumatologic or autoimmune disorders, neurological or neuromuscular disorders, cirrhosis and liver diseases, active neoplasms, obesity, active tuberculosis, haemoglobinopathies, pregnancy, malnutrition and prematurity in children <5 years),^9 11^ influenza vaccination status (administered >14 days before symptoms started was considered as immunised, otherwise unimmunised),^11^ antiviral drug usage before or during hospitalisation,^11^ and sex (male/female).^9^ We also included income group at the country level by World Bank in 2022^19^ due to their association with disease severity^9^ and enrolment during the epidemic period (defined by country as ≥5% influenza positivity for at least two consecutive weeks per WHO FluNet data^20^ considering its impact on hospital capacity^21^ and the infecting viruses. Their associations with influenza subtypes and clades were evaluated using χ^2^ tests. Influenza subtypes and clades information was derived from the GISAID metadata, with additional classifications of HA protein sequences performed for unassigned clade information using Nextclade (Aksamentov *et al*; https://clades.nextstrain.org).^22^ Reference sequences used for Nextclade were the season vaccine strains, A/Darwin/6/2021 (EPI1857216) for A(H3N2) and A/Wisconsin/588/2019 (MW626062) for A(H1N1)pdm09.

Mixed-effects logistic regression models assessed the association between virus subtype or clade and disease severity, incorporating study site as a random effect to account for site-level variation and all other explanatory variables listed above, together with the virus subtype or clade, as fixed effects. Adjusted ORs (aORs) and 95% CIs from the full models were calculated using the Wald method. Random intercepts representing study site–level variabilities were extracted, and corresponding CIs were approximated using the conditional SD. Twelve models were evaluated, consisting of one subtype-level analysis and two clade-level analyses (A(H1N1)pdm09 and A(H3N2)) applied to each of the four outcome variables. Model fit and potential misspecifications were diagnosed using the DHARMa (Diagnostics for Hierarchical Regression Models) package, which employs a simulation-based approach to create interpretable scaled residuals.^23^ This method helped in identifying issues such as overdispersion, zero-inflation and residual autocorrelation, ensuring the models’ assumptions were met adequately. For this diagnostic, we used 5000 simulations to ensure robust and reliable assessment of model assumptions.

Model robustness was validated through several sensitivity analyses to ensure the reliability of the findings. First, we evaluated the impact of different methods for handling missing data on the composite severity definitions. Comparisons were made between the primary models that included patients with complete severity data and patients with at least one severity variable coded as ‘yes’, and alternative models that included only patients with complete data or those with missing data and variables recorded as ‘no’. Second, we compared the analysis of the primary dataset with datasets in which missing explanatory variables (age, vaccination status and antiviral usage) have been replaced by one of three different methods: multiple imputation with the ‘mice’ package (details in supplement); missing_NO dataset—replacement of missing values by their reference categories (ie, 18–64 age group, no influenza vaccination and no antiviral usage) and missing_Yes dataset—replacement of missing values by their highest or ‘yes’ categories (≥65 age group, influenza vaccination and antiviral usage). Thirdly, the improvement in model fit by adding clinical and demographic covariates was assessed by comparing the primary mixed-effects models against simpler mixed-effects models that included only viral genetic information as a fixed variable and study site as random effect. Fourthly, we compared the primary mixed-effects models, which treated study site as a random effect, to fixed-effects regression models where study site was treated as a fixed effect.

The performances of the full primary mixed-effects models were quantitatively compared with the simpler mixed-effects models and fixed-effects models using the Akaike Information Criterion (AIC) and Bayesian Information Criterion (BIC). Additionally, for each analysis, comparisons between the simpler mixed-effects models and the full primary mixed-effects models were conducted using a log-likelihood ratio test (LRT).

All statistical analyses were performed using R statistical software.

### Patient and public involvement statement

None.

## Results

**Figure 1:**
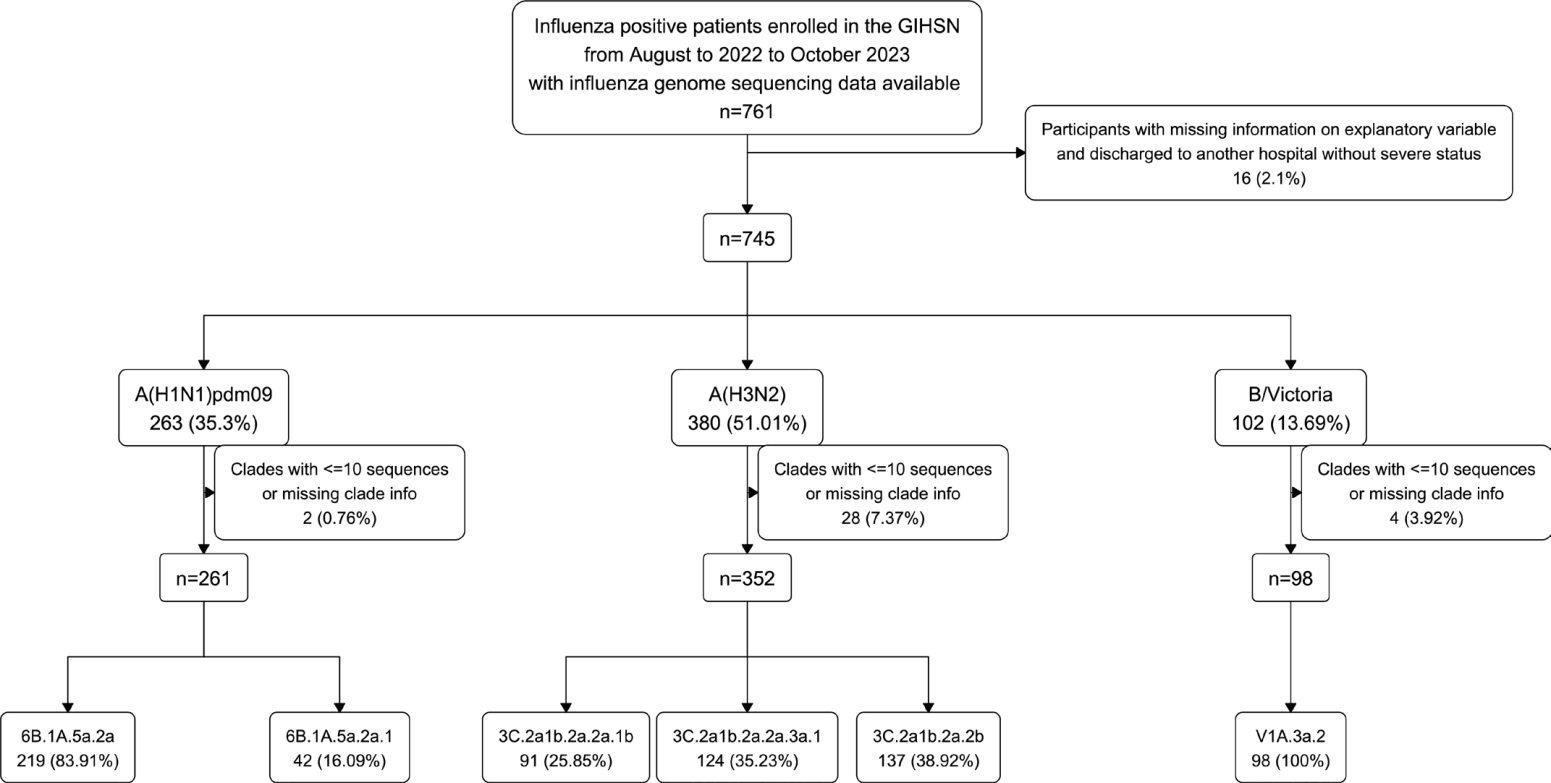
Flowchart of patient inclusion and exclusion criteria for analysis of genetic information and severity among hospitalised influenza patients enrolled in the Global Influenza Hospital Surveillance Network (GIHSN).

There were 761 influenza sequences available on GISAID collected from patients enrolled in GIHSN from August 2022 to October 2023. The samples were collected from 15 study sites across 14 countries from both hemispheres (online supplemental figure 1). Missing information was observed in 14 patients in total. Specifically, one patient was missing age data, 13 patients lacked influenza vaccination status, and five patients had no record of antiviral usage. These patients were more likely to have incomplete severity data compared with other patients (online supplemental table 1). After excluding these 14 patients and two additional patients who were discharged to another hospital without a severe status, 745 patients were included in the main analysis. These patients comprised 263 infected with A(H1N1)pdm09, 380 with A(H3N2), and 102 with B/Victoria (figure 1). For the 263 patients infected with A(H1N1)pdm09 viruses, 83.9% belonged to clade 6B.1A.5a.2a (5a.2a), 16.0% to 6B.1A.5a.2a.1 (5a.2a.1), one to 6B.1A.5a.1 (5a.1) and in one clade information was missing (figure 1 and online supplemental figure 2). Patients infected with 5a.1 or unknown clades were excluded from clade-level analyses. The 380 patients infected with A(H3N2) viruses were predominantly from clades 3C.2a1b.2a.2a.1b (2a.1b; 24.3%), 3C.2a1b.2a.2a.3a.1 (2a.3a.1; 33.1%), and 3C.2a1b.2a.2b (2b; 36.5%). Other minor clades reported were 3C.2a1b.2a.2c (n=5), 3C.2a1b.2a.2a.1 (n=8), and 3C.2a1b.2a.2a.3 (n=10), and these were excluded from clade-level analyses. Clade-level analysis was not conducted for the B/Victoria lineage as all patients had the same clade V1A.3a.2. The clades reported for A(H1N1)pdm09 and A(H3N2) subtypes varied across study sites (online supplemental figure 3). For subtype-level analyses of the 3-variable and 4-variable definition outcomes, one study site (Russia-Moscow) was excluded, as it contained only a single eligible patient. Similarly, the clade-level analysis for A(H3N2) across all outcomes excluded one site (Türkiye) due to only two patients being eligible.

Among the included patients 44.6% (n=332) were <5 years old, 21.6% (n=161) were aged 5–17 years, 17.9% (n=133) were 18–64 years, 16.0% (n=119) were ≥65 years old, and 52.2% (n=389) were male. At least one chronic medical condition was reported in 33.2% (n=247) of patients, only 5.8% (n=43) had been vaccinated against influenza, and antiviral usage was reported in 32.1% (n=239). Overall, 44.7% (n=333) of the patients were from high income countries, 35.0% (n=261) were from upper-middle income countries, and the rest were from lower-middle income countries; the majority (71.3%) of the sequenced viruses were collected during the epidemic periods. Differences across influenza subtypes were observed in patients’ age distribution, antiviral use, time of enrolment in relation to the epidemic period and country income level (table 1).

Among patients infected with A(H1N1)pdm09, differences among those with clade 5a.2a.1 or clade 5a.2a were detected in relation to age distribution, presence of chronic medical conditions and country income level (table 2).

For patients with the three main clades of A(H3N2) infections, there were differences in patients’ age distribution, antiviral usage, time of enrolment in relation to the epidemic period, presence of risk factors and country income level (table 3).

Overall, 6.2% of patients were admitted to ICU, 32.9% received oxygen supplementation, 9.3% met the 3-variable severity definition, and 36.9% the 4-variable definition (table 1 and online supplemental figure 4). There were no differences in the proportion of patients meeting the different severity definitions across the different influenza subtypes. Among A(H1N1)pdm09 viruses, clade 5a.2a.1 was detected in a higher proportion of patients needing oxygen supplementation and meeting the 4-variable definition (table 2 and online supplemental figure 4). The percentages of patients meeting the different severity definitions were similar across the three main clades of A(H3N2) subtype (table 3 and online supplemental figure 4).

**Table 1:** Patient characteristics by subtype

	A(H1N1)pdm09 (n=263)	A(H3N2) (n=380)	B/Victoria (n=102)	Total (n=745)	P value
Age groups in years					<0.001
<5	85 (32.3%)	207 (54.5%)	40 (39.2%)	332 (44.6%)	
5–17	67 (25.5%)	56 (14.7%)	38 (37.3%)	161 (21.6%)	
18–64	62 (23.6%)	52 (13.7%)	19 (18.6%)	133 (17.9%)	
≥65	49 (18.6%)	65 (17.1%)	5 (4.9%)	119 (16.0%)	
Sex					0.948
Female	124 (47.1%)	182 (47.9%)	50 (49.0%)	356 (47.8%)	
Male	139 (52.9%)	198 (52.1%)	52 (51.0%)	389 (52.2%)	
Presence of underlying medical conditions					0.539
No	169 (64.3%)	259 (68.2%)	70 (68.6%)	498 (66.8%)	
Yes	94 (35.7%)	121 (31.8%)	32 (31.7)	247 (33.2%)	
Influenza vaccination 14 days before symptoms					0.072
No	242 (92.0%)	360 (94.7%)	100 (98.0%)	702 (94.2%)	
Yes	21 (8.0%)	20 (5.3%)	2 (2.0%)	43 (5.8%)	
Usage of antiviral drugs before or during hospitalisation					<0.001
No	157 (59.7%)	285 (75.0%)	64 (62.7%)	506 (67.9%)	
Yes	106 (40.3%)	95 (25.0%)	38 (37.3%)	239 (32.1%)	
Enrolment during epidemic period					0.001
No	81 (30.8%)	119 (31.3%)	14 (13.7%)	214 (28.7%)	
Yes	182 (69.2%)	261 (68.7%)	88 (86.3%)	531 (71.3%)	
Income level					<0.001
High income	176 (66.9%)	108 (28.4%)	49 (48.0%)	333 (44.7%)	
Upper middle income	39 (14.8%)	182 (47.9%)	40 (39.2%)	261 (35.0%)	
Lower middle income	48 (18.3%)	90 (23.7%)	13 (12.7%)	151 (20.3%)	
Clades					
5a.1	1 (0.4%)	–	–	1 (0.1%)	
5a.2a	219 (83.6%)	–	–	219 (29.8%)	
5a.2a.1	42 (16.0%)	–	–	42 (5.7%)	
2a.1b	–	91 (24.3%)	–	91 (12.4%)	
2a.3a.1	–	124 (33.1%)	–	124 (16.9%)	
2b	–	137 (36.5%)	–	137 (18.6%)	
Others (H3N2)	–	23 (6.1%)	–	23 (3.1%)	
V1A.3a.2	–	–	98 (100.0%)	98 (13.3%)	
Unassigned	1	5	4	10	
Severity (4-variable definition)					0.076
No	146 (66.7%)	201 (59.1%)	56 (70.0%)	403 (63.1%)	
Yes	73 (33.3%)	139 (40.9%)	24 (30.0%)	236 (36.9%)	
Missing	44	40	22	106	
Severity (3-variable definition)					0.34
No	216 (88.5%)	337 (91.6%)	78 (92.9%)	631 (90.7%)	
Yes	28 (11.5%)	31 (8.4%)	6 (7.1%)	65 (9.3%)	
Missing	19	12	18	49	
Severity (ICU admission)					0.24
No	237 (91.9%)	352 (95.1%)	82 (94.3%)	671 (93.8%)	
Yes	21 (8.1%)	18 (4.9%)	5 (5.7%)	44 (6.2%)	
Missing	5	10	15	30	
Severity (Oxygen supplementation)					0.119
No	150 (69.8%)	220 (63.8%)	65 (73.9%)	435 (67.1%)	
Yes	65 (30.2%)	125 (36.2%)	23 (26.1%)	213 (32.9%)	
Missing	48	35	14	97	
Severity (Mechanical Ventilation)					0.765
No	235 (95.9%)	358 (96.5%)	82 (97.6%)	675 (96.4%)	
Yes	10 (4.1%)	13 (3.5%)	2 (2.4%)	25 (3.6%)	
Missing	18	9	18	45	
Severity (Death in hospital)					0.874
No	256 (97.3%)	372 (97.9%)	100 (98.0%)	728 (97.7%)	
Yes	7 (2.7%)	8 (2.1%)	2 (2.0%)	17 (2.3%)	

P values were derived from a χ2 test of the contingency table between patients’ characteristics and viral subtypes.

ICU, intensive care unit.

**Table 2:** Patient characteristics by clade in subtype A(H1N1)pdm09

	5a.2a (n=219)	5a.2a.1 (n=42)	Total (n=261)*	P value
Age groups in years				0.004
<5	75 (34.2%)	9 (21.4%)	84 (32.2%)	
5–17	57 (26.0%)	10 (23.8%)	67 (25.7%)	
18–64	55 (25.1%)	7 (16.7%)	62 (23.8%)	
≥65	32 (14.6%)	16 (38.1%)	48 (18.4%)	
Sex				0.644
Female	101 (46.1%)	21 (50.0%)	122 (46.7%)	
Male	118 (53.9%)	21 (50.0%)	139 (53.3%)	
Presence of underlying medical conditions				0.013
No	148 (67.6%)	20 (47.6%)	168 (64.4%)	
Yes	71 (32.4%)	22 (52.4%)	93 (53.3%)	
Influenza vaccination 14 days before symptoms				0.105
No	204 (93.2%)	36 (85.7%)	240 (92.0%)	
Yes	15 (6.8%)	6 (14.3%)	21 (8.0%)	
Usage of antiviral drugs before or during hospitalisation				0.176
No	134 (61.2%)	21 (50.0%)	155 (59.4%)	
Yes	85 (38.8%)	21 (50.0%)	106 (40.6%)	
Enrolment during epidemic period				0.437
No	65 (29.7%)	15 (35.7%)	80 (30.7%)	
Yes	154 (70.3%)	27 (64.3%)	181 (69.3%)	
Income level				0.036
High income	144 (65.8%)	31 (73.8%)	175 (67.0%)	
Upper middle income	30 (13.7%)	9 (21.4%)	39 (14.9%)	
Lower middle income	45 (20.5%)	2 (4.8%)	47 (18.0%)	
Severity (4-variable definition)				0.004
No	132 (70.2%)	13 (43.3%)	145 (66.5%)	
Yes	56 (29.8%)	17 (56.7%)	73 (33.5%)	
Missing	31	12	43	
Severity (3-variable definition)				0.096
No	180 (90.0%)	34 (81.0%)	214 (88.4%)	
Yes	20 (10.0%)	8 (19.0%)	28 (11.6%)	
Missing	19	0	19	
Severity (ICU admission)				0.339
No	198 (92.5%)	37 (88.1%)	235 (91.8%)	
Yes	16 (7.5%)	5 (11.9%)	21 (8.2%)	
Missing	5	0	5	
Severity (Oxygen supplementation)				0.032
No	135 (72.2%)	14 (51.9%)	149 (69.6%)	
Yes	52 (27.8%)	13 (48.1%)	65 (30.4%)	
Missing	32	15	47	
Severity (Mechanical Ventilation)				0.005
No	196 (97.5%)	37 (88.1%)	233 (95.9%)	
Yes	5 (2.5%)	5 (11.9%)	10 (4.1%)	
Missing	18	0	18	
Severity (Death in hospital)				0.24
No	212 (96.8%)	42 (100.0%)	254 (97.3%)	
Yes	7 (3.2%)	0 (0.0%)	7 (2.7%)	

P values were derived from a χ2 test of the contingency table between patients’ characteristics and A(H1N1)pdm09 clades.

* Clade 6B.1A.5a.1 (5a.1) detected in one patient and another patient without clade information was excluded from this total.

ICU, intensive care unit.

**Table 3:** Patient characteristics by clade in subtype A (H3N2)

	2a.1b (n=91)	2a.3a.1 (n=124)	2b (n=137)	Total (n=352)*	P value
Age groups in years					<0.001
<5	42 (46.2%)	93 (75.0%)	60 (43.8%)	195 (55.4%)	
5–17	14 (15.4%)	13 (10.5%)	28 (20.4%)	55 (15.6%)	
18–64	23 (25.3%)	11 (8.9%)	17 (12.4%)	51 (14.5%)	
≥65	12 (13.2%)	7 (5.6%)	32 (23.4%)	51 (14.5%)	
Sex					0.539
Female	39 (42.9%)	59 (47.6%)	69 (50.4%)	167 (47.4%)	
Male	52 (57.1%)	65 (52.4%)	68 (49.6%)	185 (52.6%)	
Presence of underlying medical conditions					0.021
No	63 (69.2%)	94 (75.8%)	82 (59.9%)	239 (67.9%)	
Yes	28 (30.8%)	30 (24.2%)	55 (40.1%)	113 (32.1%)	
Influenza vaccination 14 days before symptoms					0.464
No	86 (94.5%)	120 (96.8%)	128 (93.4%)	334 (94.9%)	
Yes	5 (5.5%)	4 (3.2%)	9 (6.6%)	18 (5.1%)	
Usage of antiviral drugs before or during hospitalisation					<0.001
No	70 (76.9%)	121 (97.6%)	80 (58.4%)	271 (77.0%)	
Yes	21 (23.1%)	3 (2.4%)	57 (41.6%)	81 (23.0%)	
Enrolment during epidemic period					<0.001
No	13 (14.3%)	50 (40.3%)	53 (38.7%)	116 (33.0%)	
Yes	78 (85.7%)	74 (59.7%)	84 (61.3%)	236 (67.0%)	
Income level					<0.001
High income	22 (24.2%)	2 (1.6%)	68 (49.6%)	92 (26.1%)	
Upper middle income	41 (45.1%)	85 (68.5%)	44 (32.1%)	170 (48.3%)	
Lower middle income	28 (30.8%)	37 (29.8%)	25 (18.2%)	90 (25.6%)	
Severity (4-variable definition)					0.998
No	49 (59.8%)	74 (60.2%)	68 (60.2%)	191 (60.1%)	
Yes	33 (40.2%)	49 (39.8%)	45 (39.8%)	127 (39.9%)	
Missing	9	1	24	34	
Severity (3-variable definition)					0.866
No	83 (91.2%)	114 (92.7%)	120 (90.9%)	317 (91.6%)	
Yes	8 (8.8%)	9 (7.3%)	12 (9.1%)	29 (8.4%)	
Missing	0	1	5	6	
Severity (ICU admission)					0.19
No	84 (92.3%)	121 (97.6%)	126 (95.5%)	331 (95.4%)	
Yes	7 (7.7%)	3 (2.4%)	6 (4.5%)	16 (4.6%)	
Missing	0	0	5	5	
Severity (Oxygen supplementation)					0.673
No	49 (60.5%)	81 (65.9%)	76 (66.1%)	206 (64.6%)	
Yes	32 (39.5%)	42 (34.1%)	39 (33.9%)	113 (35.4%)	
Missing	10	1	22	33	
Severity (Mechanical Ventilation)					0.056
No	90 (98.9%)	121 (97.6%)	124 (93.2%)	335 (96.3%)	
Yes	1 (1.1%)	3 (2.4%)	9 (6.8%)	13 (3.7%)	
Missing	0	0	4	4	
Severity (Death in hospital)					0.632
No	89 (97.8%)	120 (96.8%)	135 (98.5%)	344 (97.7%)	
Yes	2 (2.2%)	4 (3.2%)	2 (1.5%)	8 (2.3%)	

P values were derived from a χ2 test of the contingency table between patients’ characteristics and A(H3N2) clades.

* Only three main clades were included in this total.

ICU, intensive care unit.

**Figure 2:**
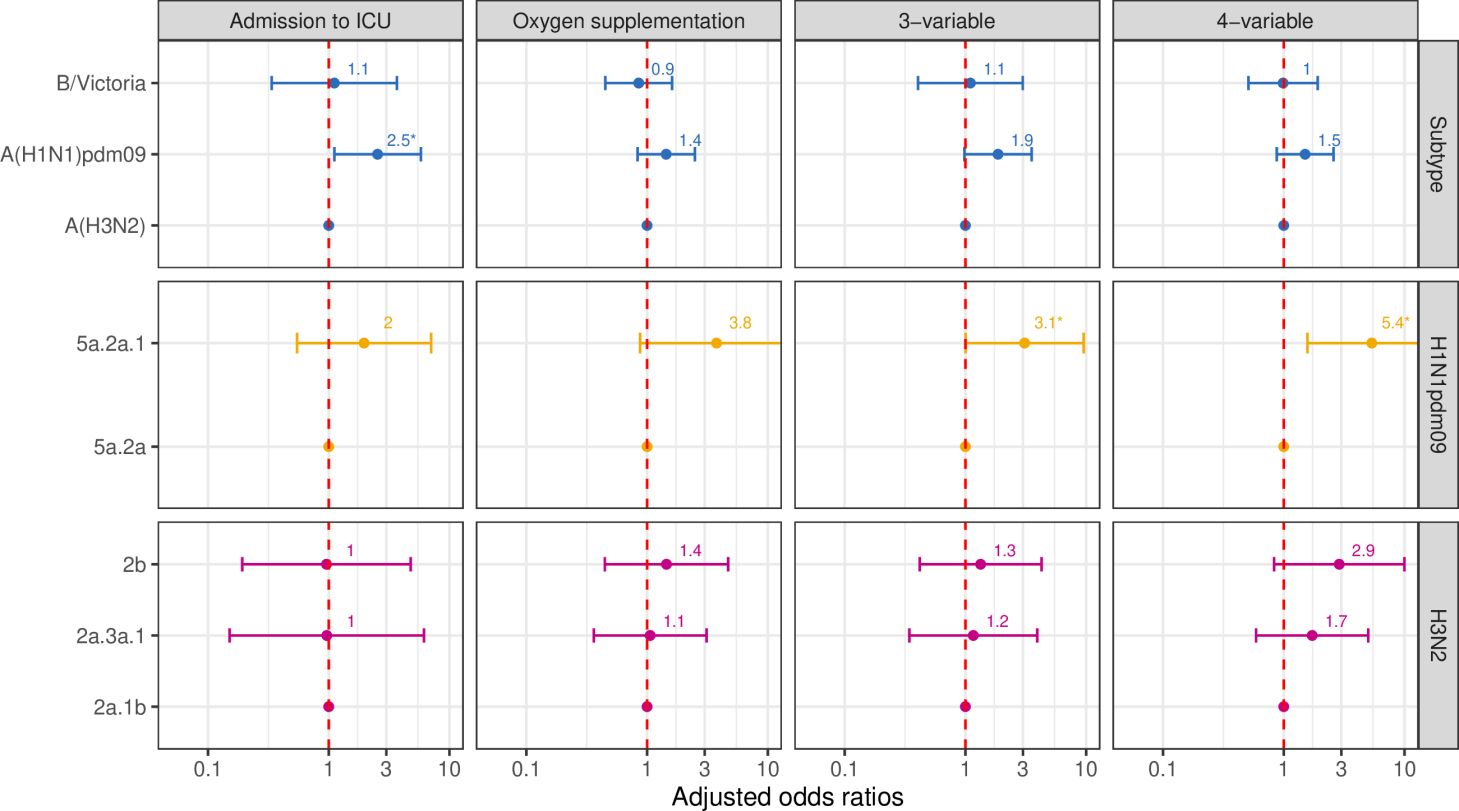
OR of viral subtypes and clades for different severity definitions among hospitalised patients. ICU, intensive care unit. * - Statistically significant results (p value <0.05).

Multivariable mixed-effects logistic regression models revealed significant differences in disease severity outcomes across influenza subtypes and clades, and summary of the key findings can be found in Box 1. A(H1N1)pdm09 infections were associated with higher odds of ICU admission (aOR 2.5, 95% CI 1.1 to 5.8, p value: 0.03) and meeting the 3-variable severity definition (aOR 1.9, 95% CI 1.0 to 3.5, p value: 0.06) compared with A(H3N2) (figure 2). B/Victoria infections showed similar odds to A(H3N2) for all severity outcomes (figure 2). Within the A(H1N1)pdm09 subtype, when comparing 5a.2a.1 clade infections with those by 5a.2a clade, higher odds were observed for the 3-variable definition (aOR 3.1, 95% CI 1.0 to 9.5, p value: 0.05) and the 4-variable definition (aOR 5.4, 95% CI 1.6 to 18.3, p value: 0.01) (figure 2). Among A(H3N2) infections, the 2b clade exhibited non-significant higher odds (aOR 2.9, 95% CI 0.8 to 10.0, p value: 0.10) compared with 2a.1b clade for the 4-variable severity definition. No differences were observed in odds for any of the severity outcomes for 2a.3a.1 clade compared with the 2a.1b clade (figure 2).

Box 1 **Summary of the key findings for analysis among hospitalised patients**

- Hospitalised patients infected with A(H1N1)pdm09 were associated with higher odds of intensive care unit admission (aOR 2.5, 95% CI 1.1 to 5.8) compared to those with A(H3N2).
- Infection with the 6B.1A.5a.2a.1 clade of A(H1N1)pdm09 showed higher odds of severity compared with the 6B.1A.5a.2a (for 3-variable definition (aOR 3.1, 95% CI 1.0 to 9.5) and 4-variable definition (aOR 5.4, 95% CI 1.6 to 18.3)).
- Infection with the 3c.2a1b.2a.2b clade of A(H3N2) showed a trend towards higher severity with a 4-variable definition (aOR 2.9, 95% CI 0.8 to 10.0) compared with 3c.2a1b.2a.2a1b.

Residual diagnostics indicated no issues with model fit, including no evidence of overdispersion, zero-inflation or residual autocorrelation (online supplemental figures 5-9 for selected models).

Sensitivity analyses, employing alternative methods for handling missing data in composite severity definitions, such as including only patients with complete information for all three or four severity criteria or including those with known information for at least one variable, in general yielded results consistent with the primary findings across all models (online supplemental figure 10). However, 5a.2a.1 clade compared with 5a.2a clade for the 4-variable definition showed lower odds ratios (aOR 3.0, 95% CI 1.1 to 7.6, p value: 0.02) than those of main analysis in complete case analysis for all severity criteria (online supplemental figure 10). While testing models with imputed covariate data or replacing unknown values with extreme values, additional patients were included (6 to 8 patients in subtype analyses, 4 to 5 patients in clade-level analyses of A(H1N1)pdm09, and 2 to 3 patients for A(H3N2)) for the different severity definitions. The results of these sensitivity analyses were similar to the main analysis, and the A(H1N1)pdm09 clade level analysis against oxygen supplementation became significant with aOR around 4.3 in these analyses (online supplemental figure 11). The simpler mixed-effects models with genetic information and study site only showed, in general, similar aORs to the main analyses, but for the A(H1N1)pdm09 clade level analyses, the aORs were closer to one for oxygen supplementation and 4-variable definition using the simpler mixed-effects models (online supplemental figure 12). The alternative models, which included study site as a fixed effect, in general produced similar aORs to the main analyses, except that the aOR for 2b clade infections compared with 2a.1b for the 4-variable definition became significant, although with wide CI, applying the fixed-effects model (aOR 4.9, 95% CI 1.4 to 18.3, p value: 0.01) (online supplemental figure 12).

Model comparisons consistently demonstrated the superiority of the full mixed-effects models over the fixed models. This was evidenced by lower AIC and BIC values and significant LRT, although a few models showed slightly lower AIC scores for fixed models (online supplemental figure 13 and online supplemental table 2). While comparing the full mixed-effects models with simpler mixed-effects models, the models for the outcomes with fewer severe patients (ICU admission and the 3-variable outcome) and A(H3N2) clade level analysis showed lower BIC values to simpler models. However, full models exhibited consistently lower AIC values and significant likelihood ratio tests (p<0.05) across all models indicating the better model fit by adding explanatory variables to the models (online supplemental figure 13 and table 2).

To better understand the variations across study sites, we examined the random intercepts. Some of these (clade level analyses with ICU admission and the 3-variable definition as outcome variables) showed a singular fit indicating negligible variance across study sites, which is identical to not including the study site in the model. All other models, however, demonstrated variations by study sites (online supplemental figure 14).

We also explored the association of the other explanatory variables, besides viral genetic information, with disease severity. Significant associations were observed in some models for the presence of underlying medical conditions, age groups, country income level and antiviral usage (online supplemental figure 15).

## Discussion

This study integrates genomic and clinical data from 14 countries to investigate associations between influenza virus types or clades and disease severity in hospitalised patients, providing a multinational perspective. It focuses on data from 2022 and 2023, a period marked by resurgence of influenza circulation following COVID-19-related public health interventions. We observed 2.5-fold higher odds of ICU admission among patients infected with A(H1N1)pdm09 compared with A(H3N2). This finding is consistent with previous studies in the USA during the 2010–2019 influenza seasons,^14^ and within the GIHSN during the 2012–2019 seasons,^9^ which reported increased severity associated with A(H1N1)pdm09. The heightened severity of A(H1N1)pdm09 could reflect its higher virulence, as demonstrated in ferret studies,^24^ and may also be influenced by immune imprinting, where early-life exposure to a specific influenza subtype shapes long-term immune responses.^25^ This is supported by recent Norwegian data from 2022 to 2023, which showed that children under five had low protective antibody titres against A(H1N1)pdm09, potentially due to reduced influenza exposure during COVID-19 lockdowns.^26^ Considering the previously reported association between the A(H1N1) subtype and disease severity in individuals born after 1957,^7^ and with children under 5 years old making up nearly half of our dataset, it is likely that our findings are also influenced by important age and cohort effects.

Within the A(H1N1)pdm09 subtype, the 5a.2a.1 clade was associated with increased odds of severe disease for composite severity definitions compared with its parent clade 5a.2a. The 5a.2a.1 clade differs from 5a.2a by specific amino acid substitutions (P137S, K142R, D260E).^27^ The Norwegian study also showed that haemagglutination inhibition titres measured in the population pre 2022–2023 influenza season were significantly lower against the 5a.2a.1 clade compared with 5a.2.^26^ This was supported by WHO data showing lower post-vaccination antibody titres in most of the human serum panels to the Northern Hemisphere 2022–2023 vaccine components, including A/Victoria/2570/2019 strain for egg-based formulations and the A/Wisconsin/588/2019 strain for cell culture-based and recombinant formulations, for both 5a.2a and 5a.2a.1 clades.^28^ Conversely, a US study conducted during the 2023–2024 season reported no significant difference in the need for oxygen supplementation between patients infected with 5a.2a versus 5a.2a.1,^29^ although that study differed from ours as it included both hospitalised and outpatient individuals. A recent pre-print reported that mutations P137S and K142R, located in both the receptor binding site and the Ca2 antigenic site, significantly contributed to immune evasion by driving antigenic drift.^30^ This immune evasion could contribute to more severe clinical outcomes by reducing host immune protection. Alternatively, the increased severity observed may reflect a direct effect of 5a.2a.1 on disease pathogenesis, independent of its ability to evade the immune system. Further research is needed to clarify the underlying mechanisms and to identify specific mutations responsible for increased virulence or immune evasion. Since February 2025, 5a.2a.1 has become the dominant A(H1N1)pdm09 clade globally, according to GISAID’s sequencing data. This clade has been competing with its parent lineage, 5a.2a, and was recommended to be included in the vaccine composition as early as 2023.^28^

For the A(H3N2) subtype, we observed a trend toward higher odds of severe outcomes (aOR 2.9) for the 2b clade compared with 2a.1b using the 4-variable severity definition. A limited sample size may have constrained statistical power, especially given the complexity of the mixed-effects models. Furthermore, the significant OR in the fixed-effects model, coupled with its wide CIs, underscored the data limitations, alongside inherent inter-site variability. The 2b clade harbours substitutions (E50K, F79V and I140K) distinguishing it from its parental clade 3C.2a1b.2a.2.^27^ Specifically, during the 2022–2023 season, the F79V substitution was identified exclusively in the 2b clade, while I140K was observed in all three main clades (2b, 2a.1b and 2a.3a.1), and E50K was shared with 2a.3a.1.^27^ A deep mutational scanning study found that the E50K mutation in influenza HA promotes immune evasion, particularly by helping the virus escape neutralising antibodies common in children.^31^ However, the detailed characteristics related to the F79V have not yet been reported, and its impact on immune evasion and/or disease severity should be explored. Genomic surveillance data from GISAID after 2023 shows a decline in 2b clade detections, with the 2a.3a.1 clade becoming predominant, highlighting the need to investigate the implications of emerging clades and their mutations. Although reduced antibody responses to the egg-adapted vaccine strain were reported for all predominant clades (but not against the cell culture-propagated strain),^28^ the very low vaccine coverage in our study population (5.8%) makes it unlikely that differences in vaccine mismatch and effectiveness alone explain the observed variation in severity.

Beyond viral genetics, our analysis also revealed that disease severity was associated with country income level, consistent with earlier findings.^9^ While adjusting for country income level accounted for some inter-site variability, residual differences suggest that local factors such as hospital capacity, clinical practices, patient management or unmeasured patient characteristics—like underlying disease severity or access to timely care—may also contribute to observed variations. However, study site-level variations were not observed for outcomes such as ICU admission and 3-variable definition, where the random effects models showed a singular fit. This suggests that for these infrequent severe events, the observed variations were largely explained by country income level and other covariates, leaving insufficient between-site variability for the random effect to be reliably estimated.

This study’s major strengths lie in its robust and comprehensive design, which significantly enhances the generalisability and reliability of the findings. The diverse socioeconomic status of the study population from different countries around the world is a key advantage, as it provides a broad representation of global health contexts and minimises the risk of findings being limited to a single region or demographic. The use of a random effects model to account for variability between study sites is a methodologically sound approach that correctly adjusts for potential clustering of outcomes within countries, thereby providing more accurate and reliable estimates of the overall effect. Furthermore, the inclusion of different sensitivity analyses strengthens the credibility of the results by demonstrating that the core findings are consistent across various assumptions and analytical approaches. Additionally, the use of a spectrum of severity outcomes—from specific endpoints like oxygen supplementation and death to a composite outcome that encompasses the full range of disease severity among hospitalised patients—offers a nuanced and comprehensive picture of the disease’s impact. This detailed approach ensures that the study captures the full clinical spectrum among hospitalised patients, providing a more complete understanding of the disease’s progression and effects. Finally, using a composite definition for severity increases the number of positive events, especially in the case of rare events in a small dataset, which enhances statistical power and the likelihood of detecting a significant association. Incorporating less severe indicators like oxygen supplementation alongside more severe outcomes, ICU admission, mechanical ventilation or death can also provide a broader perspective on the spectrum of illness which requires critical or heightened medical care; however, it is crucial to interpret the findings carefully.

This study underscores the importance of the GIHSN for monitoring the evolution and impact of influenza viruses globally. Its robust and harmonised approaches to clinical and genomic data collection across diverse settings enable the uncovering of novel insights that transcend local contexts. This capacity is essential for timely risk assessment and supports both public health and clinical decision-making, which includes the potential for informing vaccine strain selection, particularly as new viral variants emerge and health systems face evolving challenges.

Our findings must be interpreted in light of several limitations. First, the analysis was restricted to hospitalised participants with available genomic data. Viral genome sequences were not obtained for all viruses since it was limited to those with low Ct values (high viral load) and due to resource constraints in some sites, it was restricted to selected specimens, which limits the generalisability of our findings to represent the full genetic diversity. Second, despite standardised protocols for participant recruitment, there were variations in surveillance across sites, including differences in hospital types and indication for oxygen supplementation or admission to ICU, which may have introduced bias. We attempted to mitigate this by including study site as a random variable in our models. However, we could not use some informative variables like antibiotic usage and co-infection with other respiratory viruses as not all study sites collected this information or tested the viruses equally. Third, although individual immunity profiles^32^ and immune responses^33^ are known to affect disease severity, corresponding antibody levels data for the patients included in our analysis were not available in the dataset, representing a limitation of the study. Fourth, the main analysis excluded patients with missing covariate data, which could introduce bias, though sensitivity analyses suggested minimal impact. Moreover, the analysis spans a single influenza season, limiting generalisability. Additionally, data on the use of non-invasive oxygen supplementation were collected only at the time of admission, and the specific protocols for its use were not standardised across study sites. This lack of continuous data and potential for site-specific variations in clinical practice could introduce a source of bias; however, our use of a random effects model helps to mitigate the variability between sites. Finally, participants with incomplete severity data were excluded, and while they were more likely to have missing covariates, our sensitivity analyses confirmed the robustness of our primary findings.

In conclusion, our study provides a comprehensive global analysis of the association between influenza subtypes and clades and clinical severity in hospitalised patients. The results suggest increased severity associated with A(H1N1)pdm09 and specifically the 5a.2a.1 clade, with a possible signal also emerging for the A(H3N2) 2b clade. These findings underscore the value of integrated genomic and clinical surveillance and the importance of further research into the role of specific mutations in shaping influenza severity.

## Supplementary material

10.1136/bmjopen-2025-111643 online supplemental file 1

## Data Availability

Data may be obtained from a third party and are not publicly available.
