# Supplementary material for: Association of influenza viral genetic information with severity markers in patients hospitalised with influenza: multicentre retrospective cohort study
Source: BMJ Open. 2026 Jan 8;16(1):e111643. doi: 10.1136/bmjopen-2025-111643 (PMC13059944; doi:10.1136/bmjopen-2025-111643)
Supplement: online supplemental file 1 [file bmjopen-16-1-s001.pdf]

Supplementary method

Multiple imputation for missing explanatory variables

Missing values for the following covariates: age (in years), influenza vaccination status, and antiviral drug usage, were addressed using multiple imputation with the ‘mice’ package in R. The algorithm imputed the missing values by targeting each variable sequentially, generating five distinct imputed datasets. For numerical variables, missing values were imputed by drawing from observed values of similar cases using predictive mean matching (pmm). For categorical variables, logistic regression (log_reg) was used to predict probabilities, from which imputed categories were drawn. Finally, the results from these five imputed datasets were analysed and combined using Rubin's rule to provide the final pooled estimates and standard errors. All variables, excluding severity indicators and influenza genotypes, were used in the imputation process.

Supplementary Figure 1

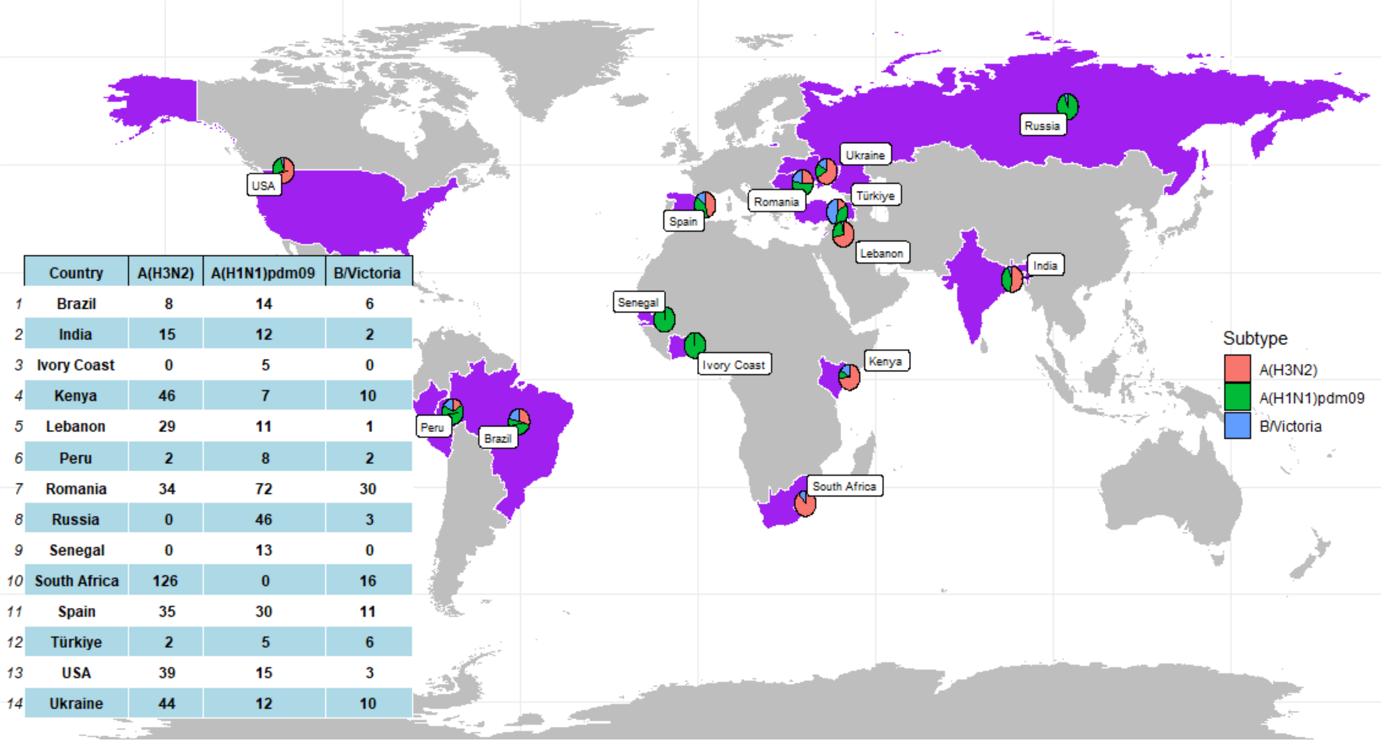

	Country	A(H3N2)	A(H1N1)pdm09	B/Victoria
1	Brazil	8	14	6
2	India	15	12	2
3	Ivory Coast	0	5	0
4	Kenya	46	7	10
5	Lebanon	29	11	1
6	Peru	2	8	2
7	Romania	34	72	30
8	Russia	0	46	3
9	Senegal	0	13	0
10	South Africa	126	0	16
11	Spain	35	30	11
12	Türkiye	2	5	6
13	USA	39	15	3
14	Ukraine	44	12	10

Supplementary Figure 1: Number of influenza sequences by subtypes and country in the GIHSN hospitals during included in this study

Supplementary Figure 2

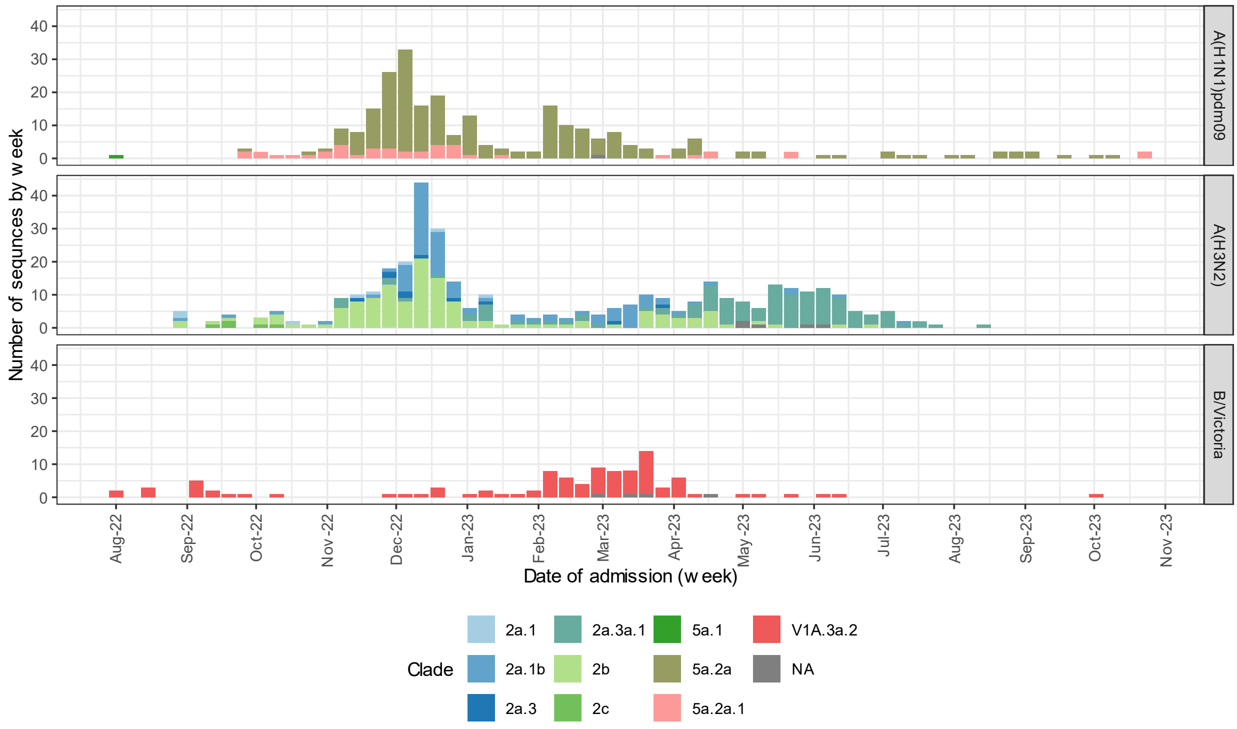

Supplementary Figure 2: Influenza clades detected for the different viral subtypes among included participants

Supplementary Figure 3

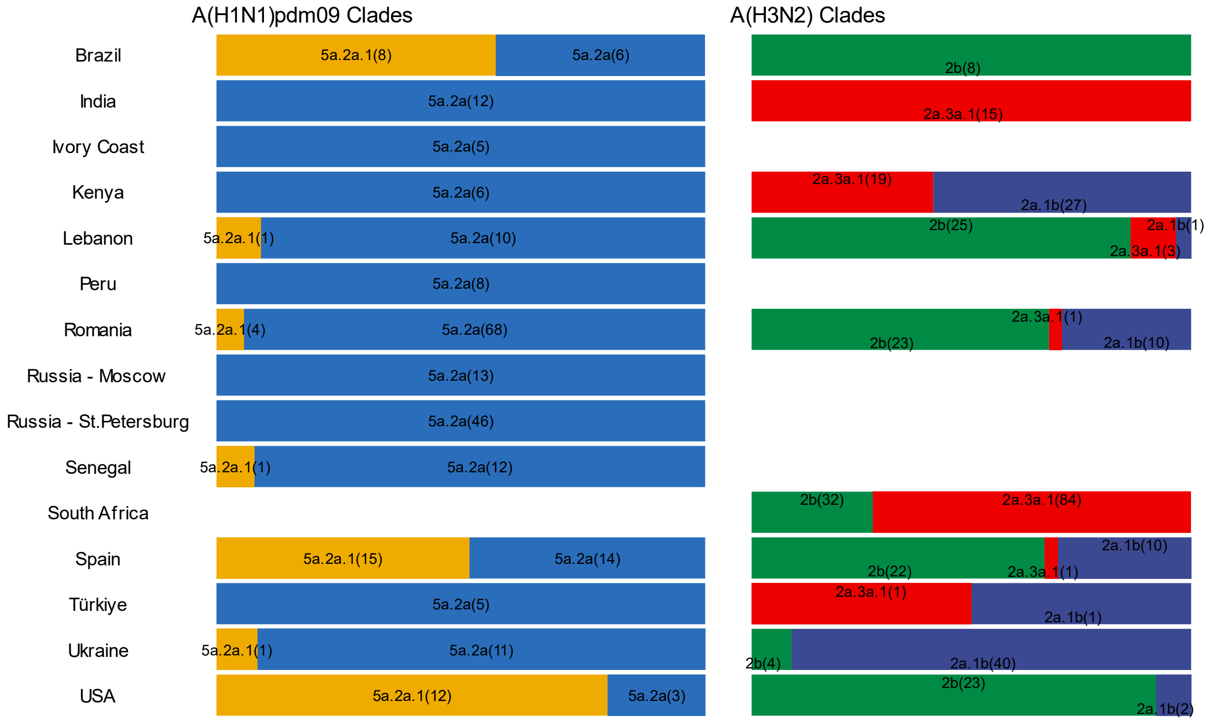

Supplementary Figure 3: Number and percentage of viruses belonging to different clades for A(H1N1)pdm09 and A(H3N2) subtypes by study site

Unassigned clades and clades with fewer than 11 sequences were excluded in the clade level analyses.

Supplementary Figure 4

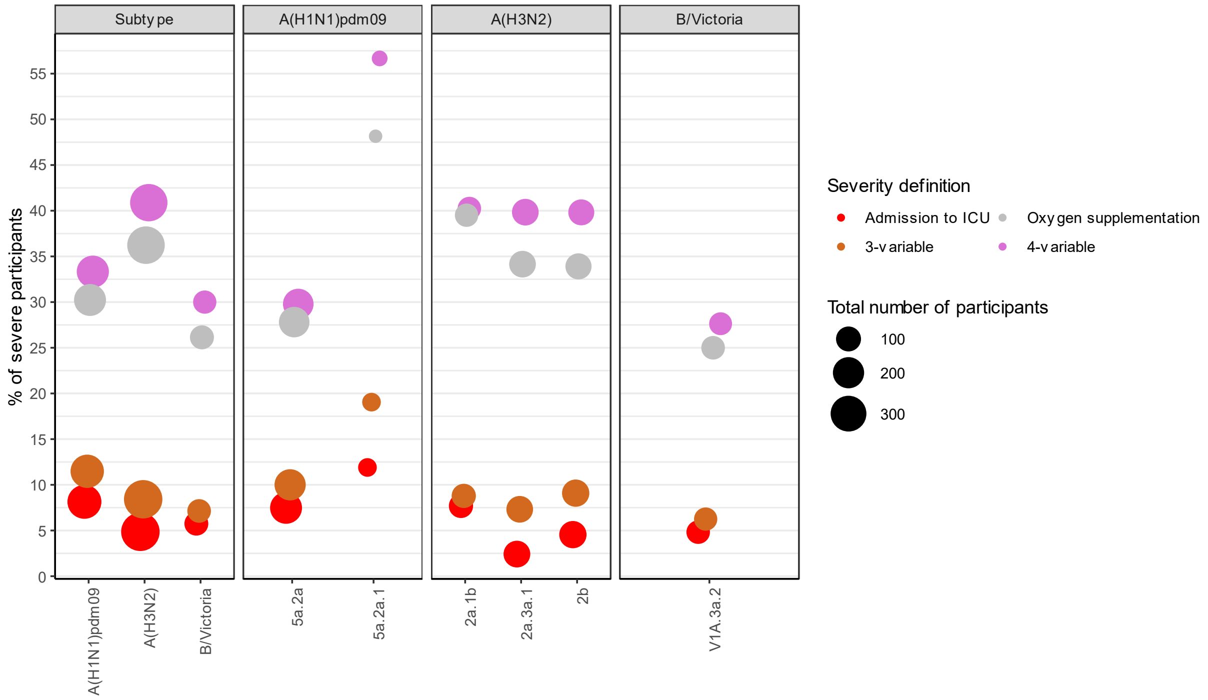

Supplementary Figure 4: Percentage of patients with severe disease by different definitions according to the viral subtypes and clades detected.

Percentages were calculated with the known information for each severity definition excluding missing and unknown cases.

Unassigned clades and clades with fewer than 11 sequences were excluded in the clade level analyses.

Supplementary Figure 5

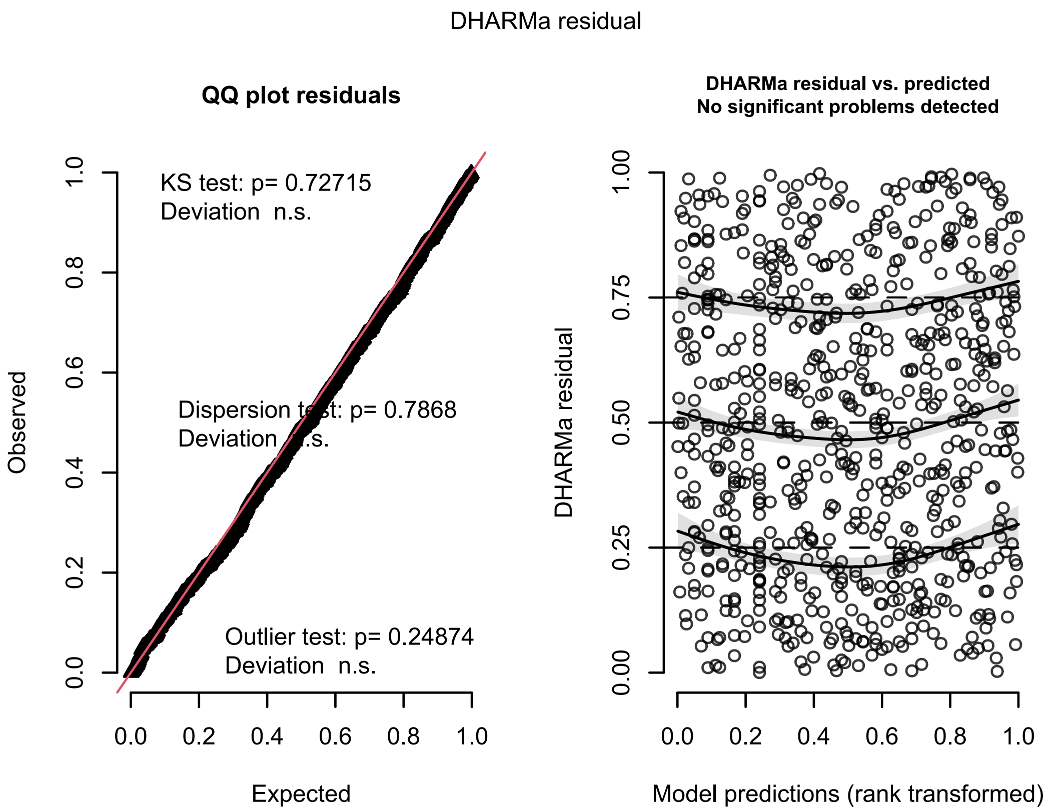

Supplementary Figure 5: Residual analysis for ICU admission model for subtype-level analysis
*KS test (p-value = 0.72), Dispersion test (p-value = 0.79), and outlier test (p-value = 0.25) on the left panel, and residual distribution by quantiles in the right panel (p-value = 0.40).

Supplementary Figure 6

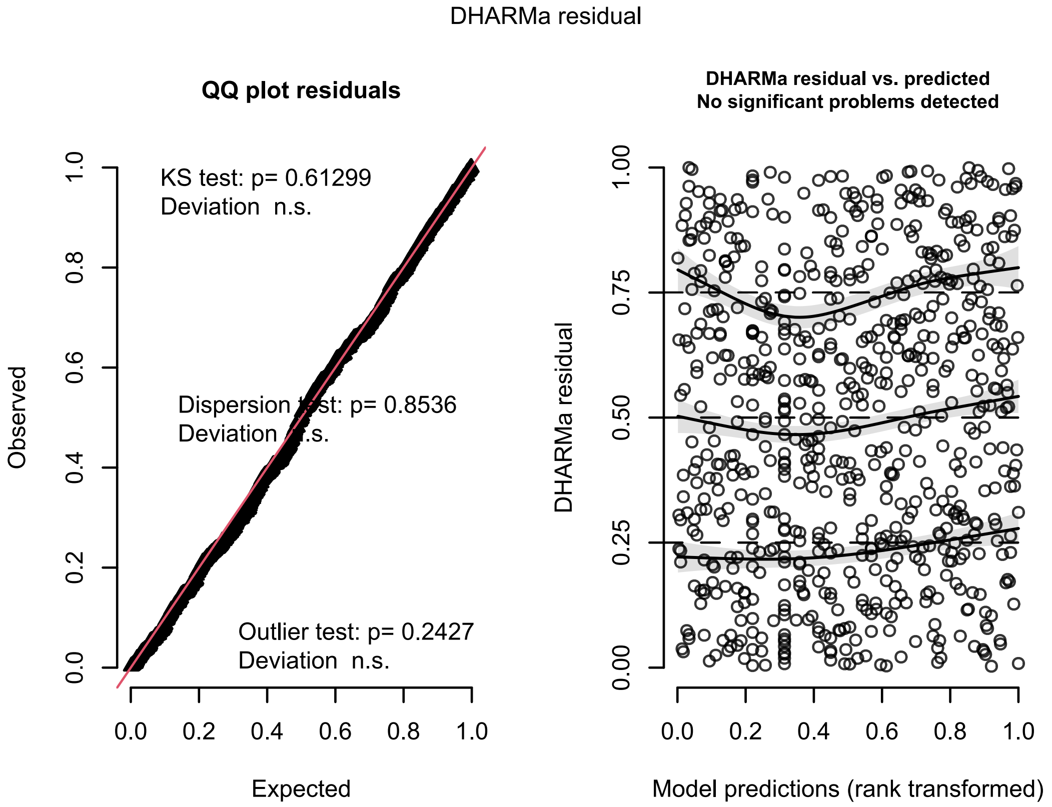

Supplementary Figure 6: Residual analysis for 3-variables composite severity model for subtype-level analysis *KS test (p-value = 0.61), Dispersion test (p-value = 0.85), and outlier test (p-value = 0.24) on the left panel, and residual distribution by quantiles in the right panel (p-value = 0.30).

Supplementary Figure 7

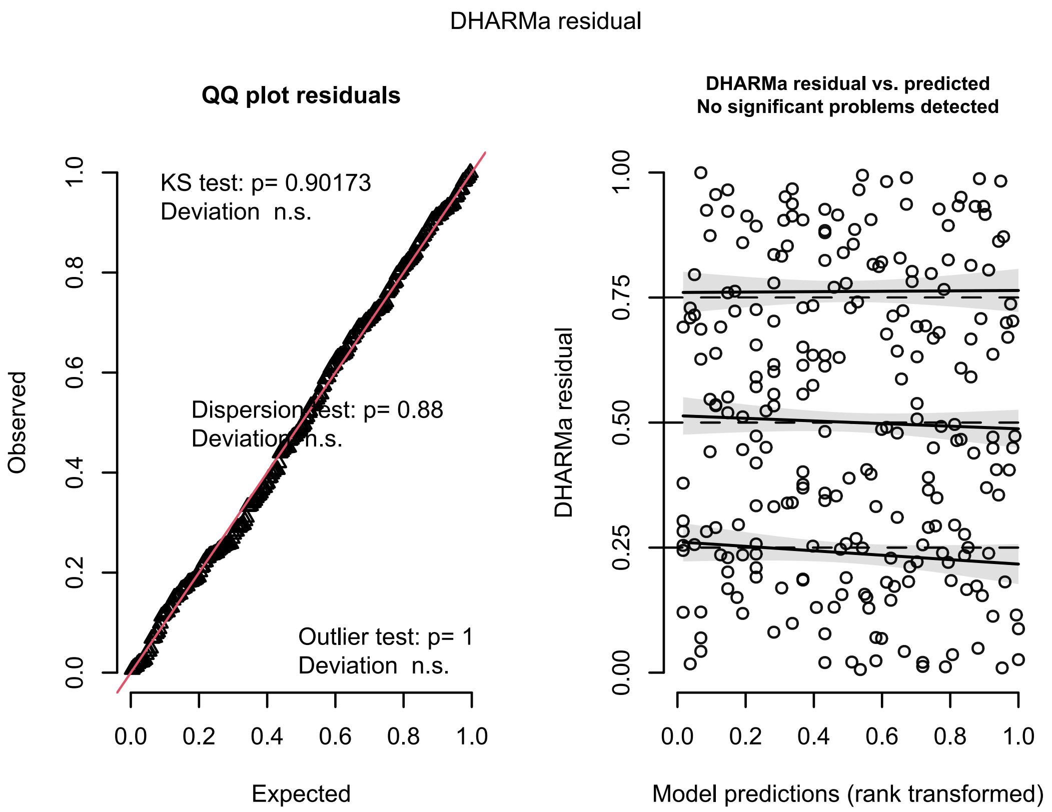

Supplementary Figure 7: Residual analysis for 3-variables composite severity model for A(H1N1)pdm09 clade-level analysis
*KS test (p-value = 0.90), Dispersion test (p-value = 0.88), and outlier test (p-value = 1) on the left panel, and residual distribution by quantiles in the right panel (p-value = 0.97)

Supplementary Figure 8

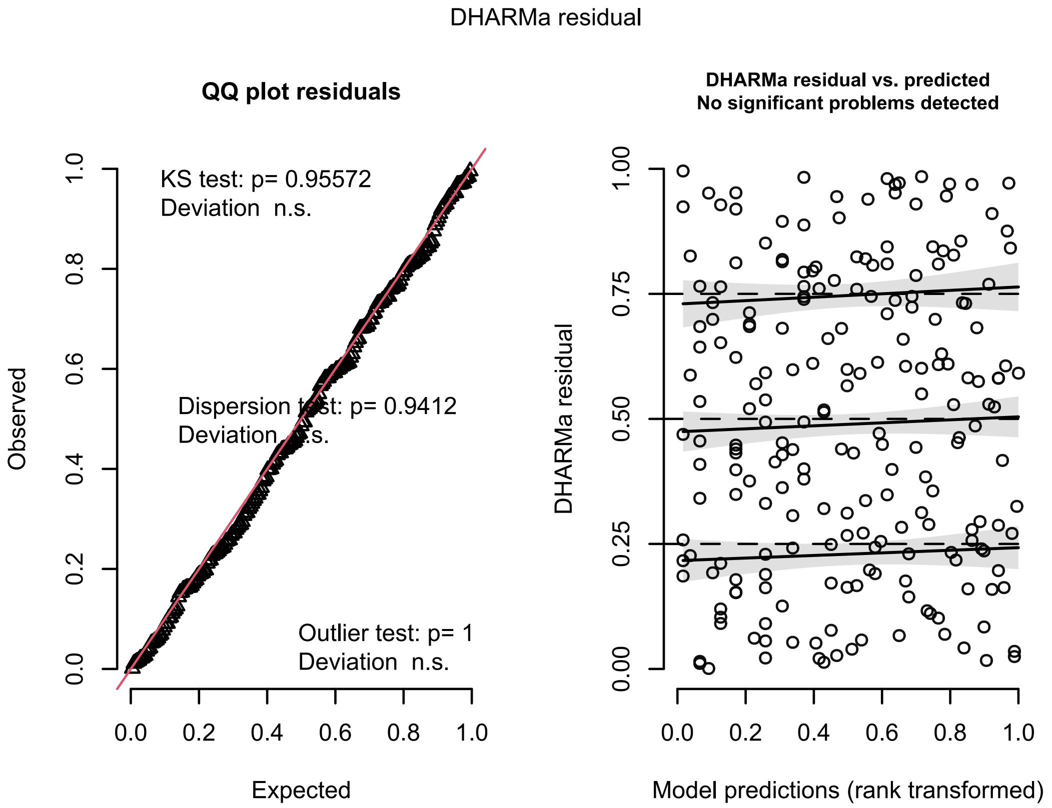

Supplementary Figure 8: Residual analysis for 4-variables composite severity model for A/H1N1pdm09 clade-level analysis

*KS test (p-value = 0.96), Dispersion test (p-value = 0.94), and outlier test (p-value = 1) on the left panel, and residual distribution by quantiles in the right panel (p-value = 0.89)

Supplementary Figure 9

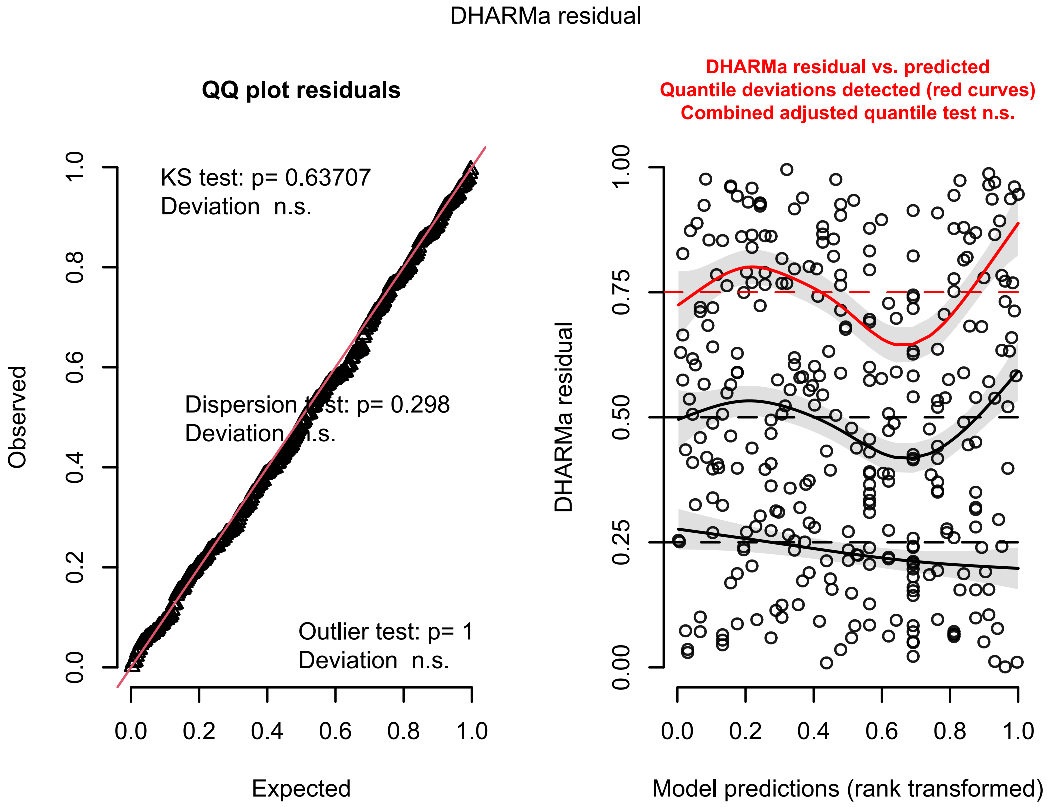

Supplementary Figure 9: Residual analysis for 4-variables composite severity model for A/H3N2 clade-level analysis *KS test (p-value = 0.64), Dispersion test (p-value = 0.29), and outlier test (p-value = 1) on the left panel, and residual distribution by quantiles in the right panel (p-value = 0.71)

Supplementary Figure 10

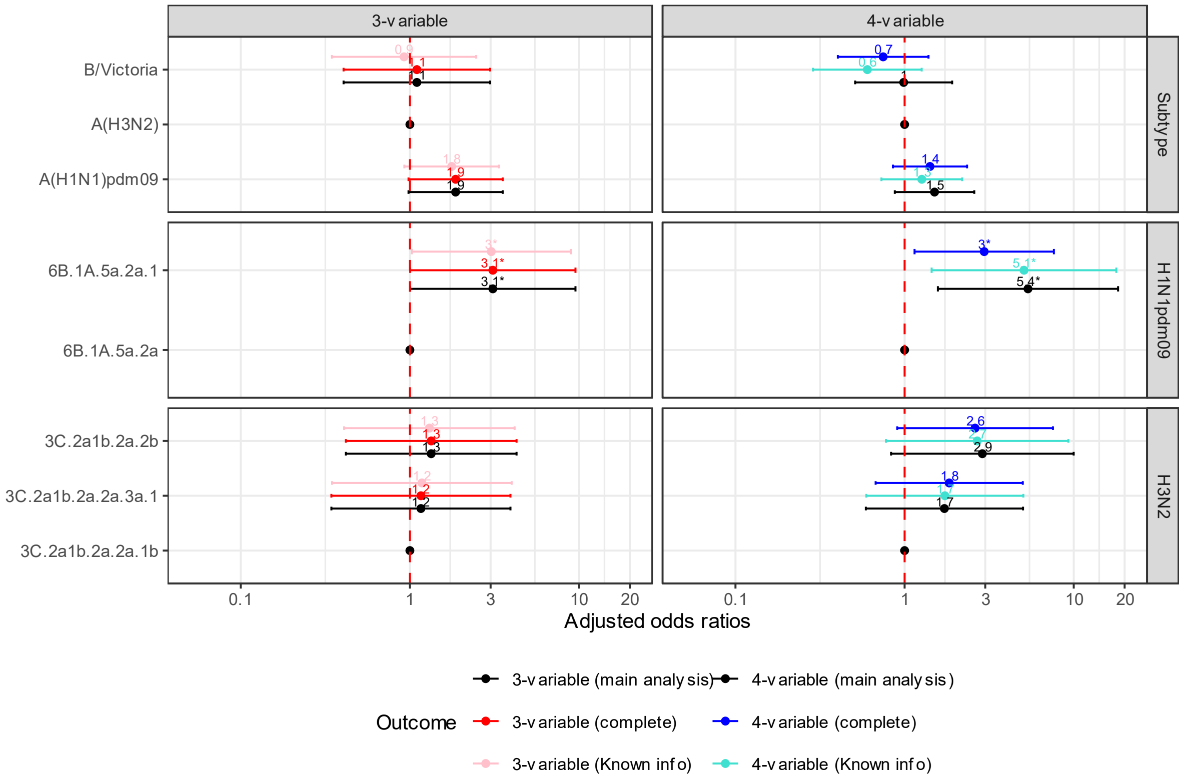

Supplementary Figure 10: Odds ratios for viral subtype and clade using different methods for handling missing data in composite severity definitions

- 3/4 variables (main analyses) - participants known to be severe or have complete information;
- 3/4 variables (complete) – participants with complete information for all severity criteria;
- 3/4 variables (Known Info) – participants with known information for at least one severity criterion)
- Mixed-effect models were adjusted for age, sex, underlying medical conditions, antiviral usage, influenza vaccination status, epidemic period, and country income level, and study site was incorporated as random effect. X-axis uses a log10 scale.
- * - Statistically significant results (p-value <0.05)

Supplementary Figure 11

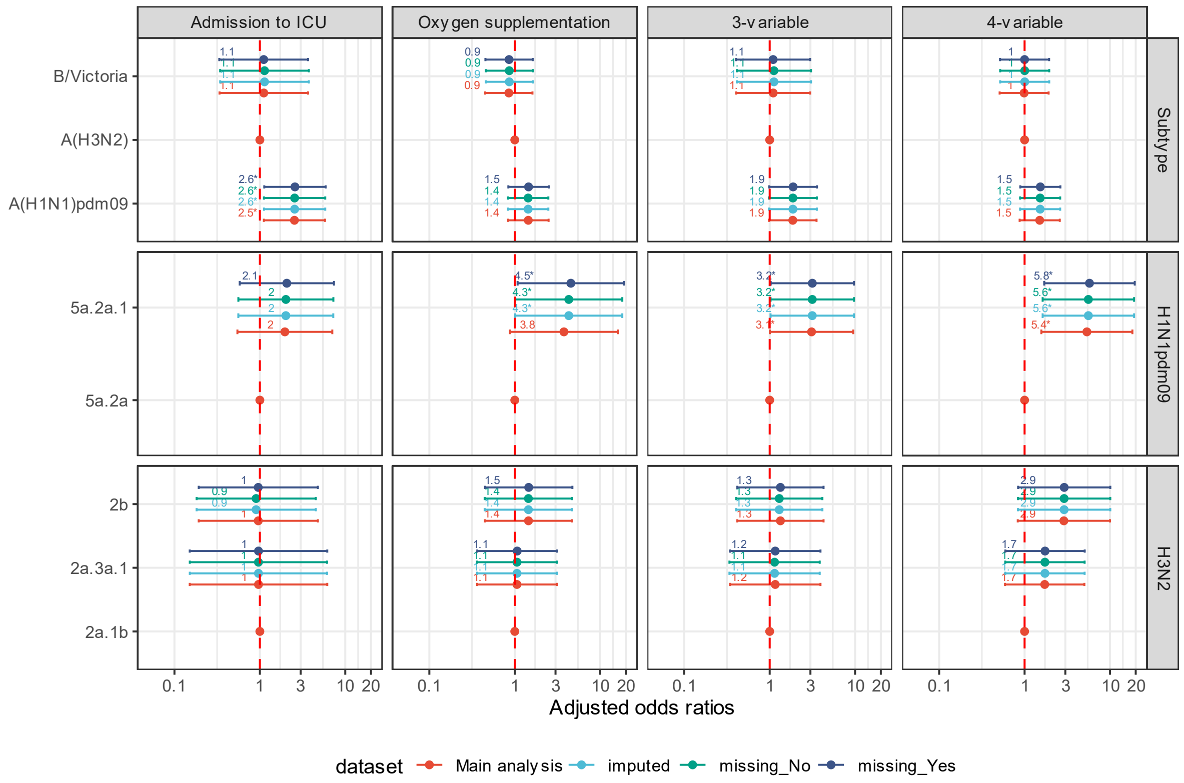

Supplementary Figure 11: Odds ratios of viral subtype and clade in the mixed-effects models with different datasets

- Main analysis - without missing data in the explanatory variables,
- imputed - analysis with imputed dataset,
- missing_No - missing data of the explanatory variables as reference category [18-64 age group, no influenza vaccination, and no antiviral usage], and
- missing_Yes - missing data of the explanatory variables as highest category [old age, influenza vaccination, and antiviral usage])
- Mixed-effect models were adjusted for age, sex, underlying medical conditions, antiviral usage, influenza vaccination status, epidemic period, and country income level, and study site was incorporated as random effect. X-axis uses a log10 scale.
- * - Statistically significant results (p-value <0.05)

Supplementary Figure 12

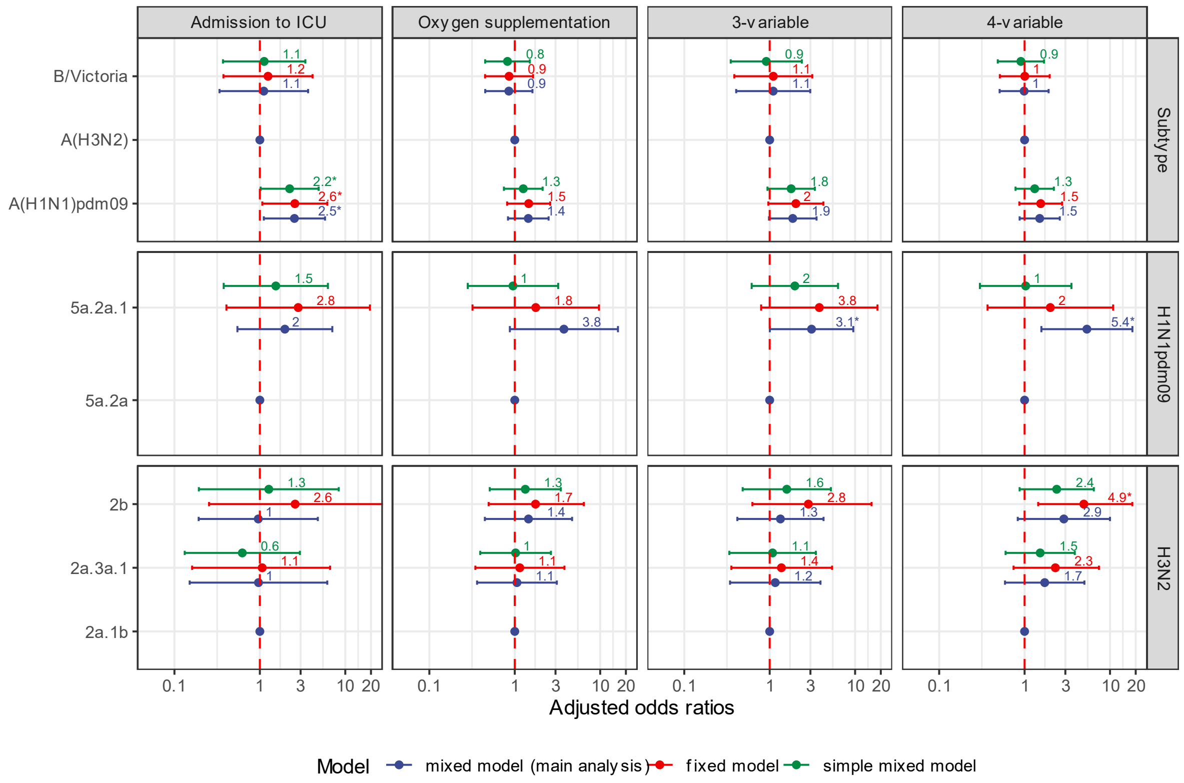

Supplementary Figure 12: Odds ratios of viral subtype and clade in the mixed-effects models (main analysis), the fixed-effects models (using study site as a fixed variable), and simple mixed model (only genetic information and the random variable - study site)

- Mixed-effect models were adjusted for age, sex, underlying medical conditions, antiviral usage, influenza vaccination status, epidemic period, and country income level, and study site was incorporated as random effect. X-axis uses a log10 scale.
- * - Statistically significant results (p-value <0.05)

Supplementary Figure 13

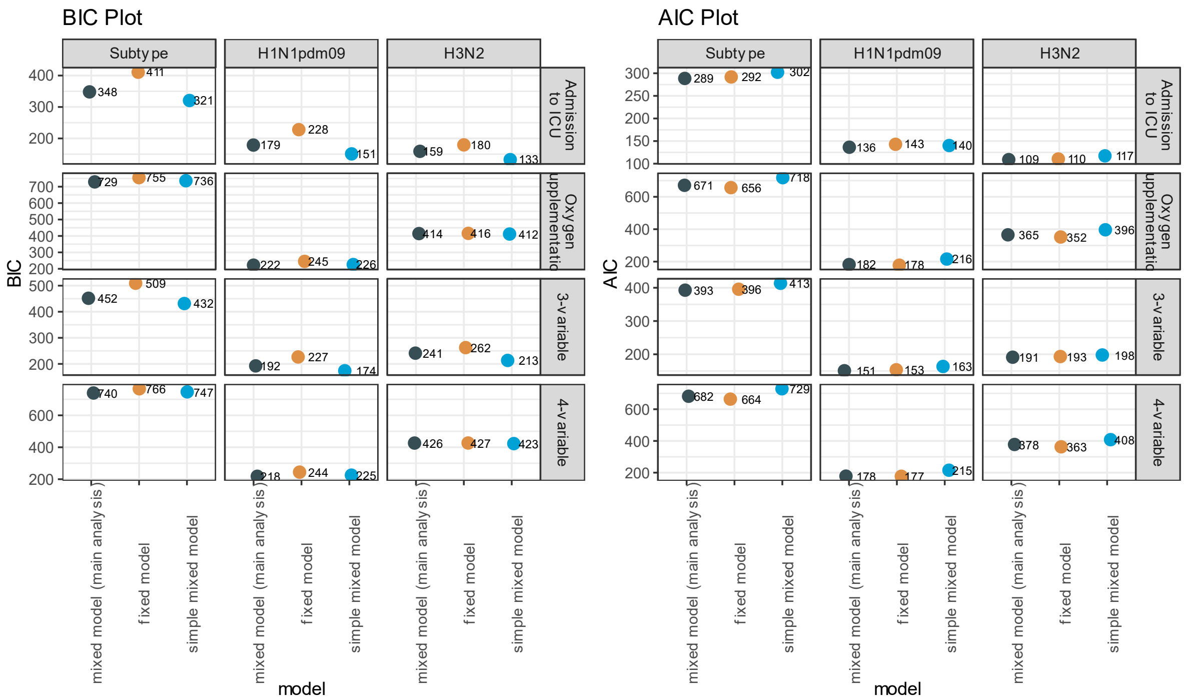

Supplementary Figure 13: Bayesian Information Criterion (BIC) and Akaike Information Criterion (AIC) values of different models - the mixed-effects models (main analysis), the fixed-effects models (using study site as a fixed variable), and simple mixed model (only genetic information and the random variable - study site)

Supplementary Figure 14

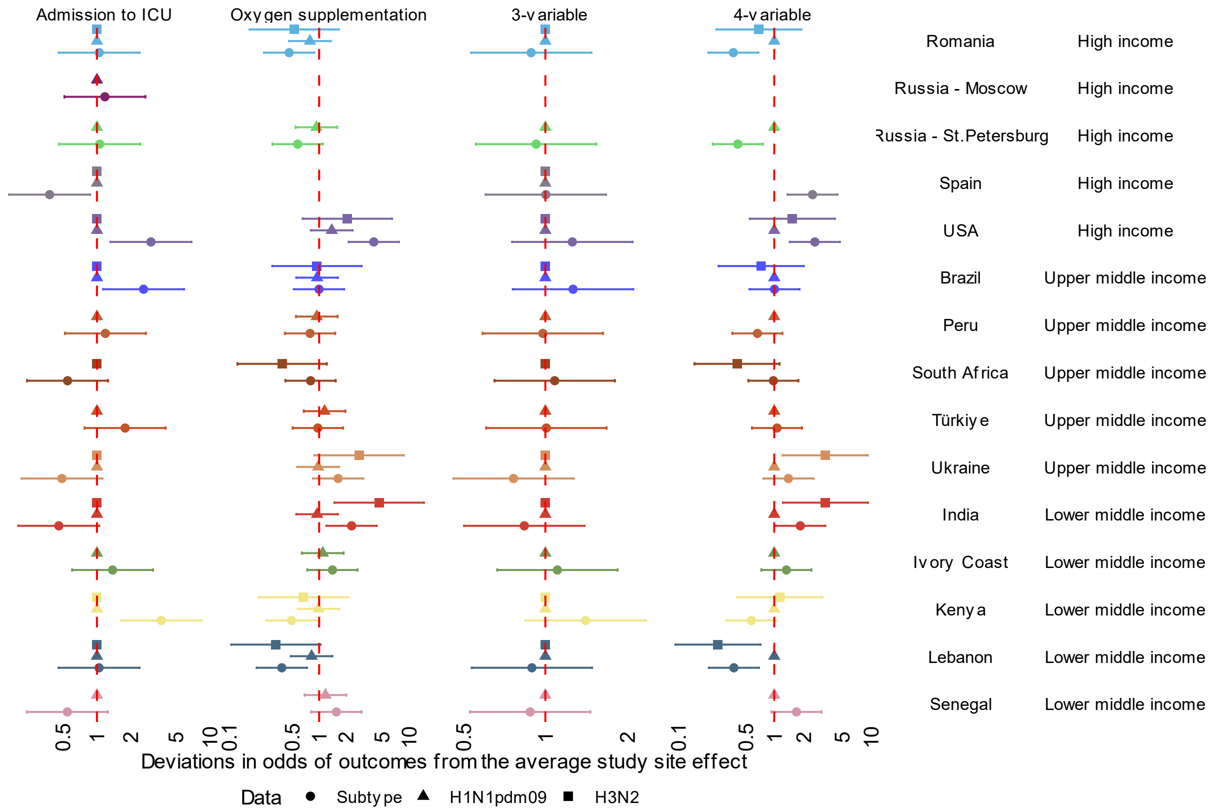

Supplementary Figure 14: Study Site-Specific Random Intercepts and Uncertainty Across Different Models (Singular fit of random effect in the model presented with odds ratio 1 by default without confidence interval.)

Supplementary Figure 15

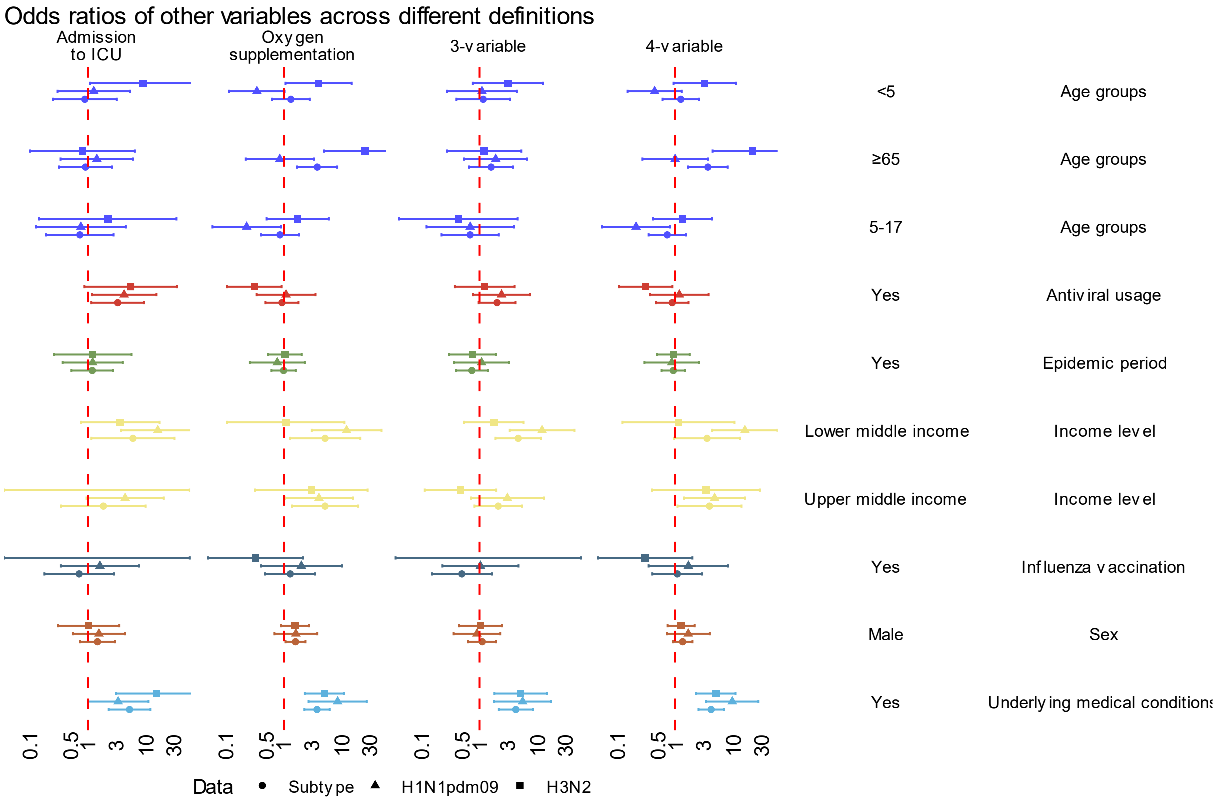

Supplementary Figure 15: Odds ratios of other variables in the mixed-effects models for different severity definitions in subtype or clade level analyses. Reference levels: 5-64 years (age group), No (antiviral usage), No (epidemic period), High income (income level), No (influenza vaccination), No (underlying medical conditions), Female (sex).

Supplementary Table 1: Missing data status for severity criteria by missingness in the explanatory variables

	**Overall (n=761)**	**No missing data in the explanatory variables (n=747)**	**Missing at least one of the explanatory variables (n=14)**
4 variables definition			
Missing	116 (15.2%)	108 (14.5%)	8 (57.1%)
3 variables definition			
Missing	58 (7.6%)	51 (6.8%)	7 (50.0%)
ICU admission			
Missing	36 (4.7%)	30 (4.0%)	6 (42.9%)
Oxygen supplementation			
Missing	106 (13.9%)	99 (13.3%)	7 (50.0%)
Death in hospital			
Missing	5 (0.7%)	0 (0%)	5 (35.7%)

Supplementary Table 2: P-values of Likelihood ratios test between primary mixed-effects models and simple mixed-effects models (genetic information and study site only). P-values <0.05 indicated the significant fit of primary mixed-effects models.

**No.**	**Dataset**	**Analysis**	**Outcome variable**	**p-value of likelihood ratios test**
**1**	Full	Subtype level	ICU admission	0.0004
**2**	Full	Subtype level	Oxygen supplimentation	< 0.0001
**3**	Full	Subtype level	3-variables definition	< 0.0001
**4**	Full	Subtype level	4-variables definition	< 0.0001
**5**	H1N1pdm09	Clade level	ICU admission	0.0156
**6**	H1N1pdm09	Clade level	Oxygen supplimentation	< 0.0001
**7**	H1N1pdm09	Clade level	3-variables definition	0.0006
**8**	H1N1pdm09	Clade level	4-variables definition	< 0.0001
**9**	H3N2	Clade level	ICU admission	0.0029
**10**	H3N2	Clade level	Oxygen supplimentation	< 0.0001
**11**	H3N2	Clade level	3-variables definition	0.0049
**12**	H3N2	Clade level	4-variables definition	< 0.0001
