# Supplementary material for: Association of influenza viral genetic information with severity markers in patients hospitalised with influenza: multicentre retrospective cohort study
Source: BMJ Open. 2026 Jan 8;16(1):e111643. doi: 10.1136/bmjopen-2025-111643 (PMC13059944; doi:10.1136/bmjopen-2025-111643)
Supplement: Supplementary file 2 [file bmjopen-2025-111643.reviewer_comments.pdf]

PEER REVIEW HISTORY

BMJ Open publishes all reviews undertaken for accepted manuscripts. Reviewers are asked to complete a checklist review form and are provided with free text boxes to elaborate on their assessment. These free text comments are reproduced below.

ARTICLE DETAILS

Title (Provisional)

Association of influenza viral genetic information with severity markers in patients hospitalized with influenza: Multicenter retrospective cohort study

Authors

Myint, Aung Pone; Shirreff, George; Baillie, Vicky; Bal, Antonin; Boutros, Celina F; Burtseva, Elena; Coulibaly, Daouda; Danilenko, Daria; Dbaibo, Ghassan; Destras, Gregory; Dia, Ndongo; Drăgănescu, Cristina Anca; Giamberardino, Heloisa I. G.; Komissarov, Andrey B; Koul, Parvaiz A.; Laguna-Torres, Victor Alberto; LeBlanc, Jason J; Mira-Iglesias, Ainara; Mironenko, Alla; Orrico-Sánchez, Alejandro; Otieno, Nancy; Săndulescu, Oana; Simon, Viviana; Sominina, Anna; Sordillo, Emilia; Tanriover, Mine Durusu; Teteriuk, Nataliia; Unal, Serhat; Van Bakel, Harm; Andrew, Melissa; Bresee, Joseph S.; Lina, Bruno; López- Labrador, F. Xavier; Ortiz, Justin; Raboni, Sonia M; Zhang, Wenqing; Chaves, Sandra S.; Cacciapaglia, Giacomo; Josset, Laurence; Chauvel, Cécile; Nunes, Marta

---

VERSION 1 - REVIEW

**Reviewer**	**1**
**Name**	**Thomas, Roger**
**Affiliation**	**University of Calgary, Family Medicine**
**Date**	**17-Oct-2025**
**COI**	**None**

Purpose

The purpose of the study is highly relevant:

“Recent studies highlight the higher rates of hospitalizations associated with A(H3N2) subtype compared with A(H1N1)pdm09 or influenza-B with some seasonal variations (5,6). Nonetheless, A/H1N1 has been associated with severe disease among those born after 1957 (7) and is often associated with more severe outcomes in hospitalized patients (8–14). Notably, patients infected with specific clades such as the A(H3N2) reassortant re3C.2a.2 had higher Influenza Severity Score during the particularly harsh 2017-2018 season in the USA

(15), underscoring the need to track the rapid genomic changes and emergent strains of influenza viruses.

Methods

“This study leverages data from the Global Influenza Hospital Surveillance Network (GIHSN), which encompasses a consortium of hospitals in more than 25 countries across diverse geographical regions (16). During the 2022-23 season (defined by the GIHSN as November through October), the GIHSN collected data from over 30,000 hospitalized patients, identifying 3,285 confirmed influenza cases (17). Notably, of these cases, 888 whole genome sequences were generated and shared via the EpiFlu database of the Global Initiative on Sharing All Influenza Data (GISAID) platform (17).”

[How does the 27% rate of obtaining whole genome sequences affect the generalisability of your study?]

“Patients were excluded from the main analysis if they had missing data on any explanatory variable, had incomplete severity information, or were transferred to another hospital without a documented severe outcome before transfer. For clade-level analyses, sequences lacking clade information were excluded from the analysis. Furthermore, clades reported in 10 or fewer patients were excluded to ensure statistical reliability and model stability. For each model, study sites with fewer than five participants were omitted”

[How many patients were excluded for each of these reasons?]

Risk factors

“Various demographic and clinical characteristics were summarized and included as explanatory variables in the model; age group (14-days before symptoms started was considered as immunized, otherwise unimmunized), antiviral drug usage before or during hospitalization, enrolment during the epidemic period (defined by country as ≥5% influenza positivity for at least two consecutive weeks per WHO FluNet data (19)), and income group at the country level by World Bank on 2022 (20).”

[This is a sensible list but not prioritised by reference to key variables which emerged in previous studies of this problem].

Analysis

[Appropriate use of methods]

Results, Discussion

The Tables, results and discussion sections are very dense (and appropriately so). Could you please summarise the key findings in a box so that Ministries of Health and those who plan vaccination campaigns can pick up the results promptly and be enabled to act?

**Reviewer**	**2**
**Name**	**Tang, Julian**
**Affiliation**	**University Hospitals of Leicester NHS Trust, Clinical Microbiology**
**Date**	**22-Oct-2025**
**COI**	**None**

The authors have performed a detailed study which includes a wide range of metadata - but the crucial host immune status is missing.

Ideally, accompanying HAI or MN titres should have been taken around the time of the viral sequence samples. Influenza vaccine status is not enough, as we do not know how individuals will respond to vaccination - and individual level responses are relevant to such clinical severity studies.

This earlier report shows the impact of pre-existing cross-reactive immunity - and can make the difference between mild disease that does not require hospitalisation vs more severe disease that does:

https://www.nejm.org/doi/pdf/10.1056/NEJMoa0906453

Indeed, we saw a lot of this variation in COVID-19 vaccine protection also.

Such immune responses at the individual level are very difficult to generalise via modelling, but admittedly, it is probably impossible to obtain these contemporaneous virus-specific antibody titres at this stage (unless the authors have archived sera for these 745 study participants). So all they can do is add a further detailed discussion about this limitation.

Unfortunately, we see this type of preferential focus on viral sequencing vs serology in many studies due to the lack of expertise/facilities to cover both angles - but without both sides, the conclusions of such 'one-sided' studies can be misleading.

VERSION 1 - AUTHOR RESPONSE

Reviewer: 1

Prof. Roger Thomas, University of Calgary

The purpose of the study is highly relevant: “Recent studies highlight the higher rates of hospitalizations associated with A(H3N2) subtype compared with A(H1N1)pdm09 or influenza-B with some seasonal variations (5,6). Nonetheless, A/H1N1 has been associated with severe disease among those born after 1957 (7) and is often associated with more severe outcomes in hospitalized patients (8–14). Notably, patients infected with specific clades such as the A(H3N2) reassortant re3C.2a.2 had higher Influenza Severity Score during the particularly harsh 2017-2018 season in the USA (15), underscoring the need to track the rapid genomic changes and emergent strains of influenza viruses.”

Authors: We thank the reviewer's comment.

Methods

"This study leverages data from the Global Influenza Hospital Surveillance Network (GIHSN), which encompasses a consortium of hospitals in more than 25 countries across diverse geographical regions (16). During the 2022-23 season (defined by the GIHSN as November through October), the GIHSN collected data from over 30,000 hospitalized patients, identifying 3,285 confirmed influenza cases (17). Notably, of these cases, 888 whole genome sequences were generated and shared via the EpiFlu database of the Global Initiative on Sharing All Influenza Data (GISAID) platform (17)."

[How does the 27% rate of obtaining whole genome sequences affect the generalizability of your study?]

Authors: We agree with the reviewer that this limitation should be acknowledge. We have now edited the discussion section to include how the low number of sequences can limit the generalizability.

"Patients were excluded from the main analysis if they had missing data on any explanatory variable, had incomplete severity information, or were transferred to another hospital without a documented severe outcome before transfer. For clade-level analyses, sequences lacking clade information were excluded from the analysis. Furthermore, clades reported in 10 or fewer patients were excluded to ensure statistical reliability and model stability. For each model, study sites with fewer than five participants were omitted"

[How many patients were excluded for each of these reasons?]

Authors: We have now detailed the number of excluded patients in the methods section and the first paragraph of the results section to complete the information that is displayed in figure 1.

Risk factors

"Various demographic and clinical characteristics were summarized and included as explanatory variables in the model; age group (14-days before symptoms started was considered as immunized, otherwise unimmunized), antiviral drug usage before or during hospitalization, enrolment during the epidemic period (defined by country as ≥5% influenza positivity for at least two consecutive weeks per WHO FluNet data (19)), and income group at the country level by World Bank on 2022 (20)."

[This is a sensible list but not prioritised by reference to key variables which emerged in previous studies of this problem].

Authors: We have now justified the inclusion of certain variables available on the dataset or variables that we produced (e.g. enrolment period) by including the respective references.

Analysis

[Appropriate use of methods]

Authors: We thank the reviewer's comment.

Results, Discussion

The Tables, results and discussion sections are very dense (and appropriately so). Could you please summarise the key findings in a box so that Ministries of Health and those who plan vaccination campaigns can pick up the results promptly and be enabled to act?

Authors: We thank the reviewer for the suggestion and we have now added table 4 as Summary of the key findings for analysis among hospitalized patients.

Reviewer: 2

Prof. Julian Tang, University Hospitals of Leicester NHS Trust

The authors have performed a detailed study which includes a wide range of metadata - but the crucial host immune status is missing.

Ideally, accompanying HAI or MN titres should have been taken around the time of the viral sequence samples. Influenza vaccine status is not enough, as we do not know how individuals will respond to vaccination - and individual level responses are relevant to such clinical severity studies.

This earlier report shows the impact of pre-existing cross-reactive immunity - and can make the difference between mild disease that does not require hospitalisation vs more severe disease that does:

https://www.nejm.org/doi/pdf/10.1056/NEJMoa0906453

Indeed, we saw a lot of this variation in COVID-19 vaccine protection also.

Such immune responses at the individual level are very difficult to generalise via modelling, but admittedly, it is probably impossible to obtain these contemporaneous virus-specific antibody titres at this stage (unless the authors have archived sera for these 745 study participants). So all they can do is add a further detailed discussion about this limitation.

Unfortunately, we see this type of preferential focus on viral sequencing vs serology in many studies due to the lack of expertise/facilities to cover both angles - but without both sides, the conclusions of such 'one-sided' studies can be misleading.

Authors: We agree with the reviewer that antibody titers would have been very informative to help predicting severity, but unfortunately, as predicted by the reviewer no blood samples were collected. We nonetheless followed the reviewer’s suggestion and added this point as the third study limitation including references.

Thank you for your time and consideration. We look forward to your response.

VERSION 2 - REVIEW

**Reviewer**	**1**
**Name**	**Thomas, Roger**
**Affiliation**	**University of Calgary, Family Medicine**
**Date**	**13-Nov-2025**
**COI**	

Thanks to the authors for their thorough changes.

**Reviewer**	**2**
**Name**	**Tang, Julian**

Affiliation **University Hospitals of Leicester NHS Trust, Clinical Microbiology**

Date **12-Nov-2025**

COI

Such studies all have similar limitations - the authors cannot do much more than acknowledge them.
